# Delayed histochemical alterations within the neurovascular unit due to transient focal cerebral ischemia and experimental treatment with neurotrophic factors

**DOI:** 10.1371/journal.pone.0174996

**Published:** 2017-04-26

**Authors:** Dominik Michalski, Roman Pitsch, Deepu R. Pillai, Bianca Mages, Susanne Aleithe, Jens Grosche, Henrik Martens, Felix Schlachetzki, Wolfgang Härtig

**Affiliations:** 1 Department of Neurology, University of Leipzig, Liebigstr. 20, Leipzig, Germany; 2 Paul Flechsig Institute for Brain Research, University of Leipzig, Liebigstr. 19, Leipzig, Germany; 3 Department of Neurology, University of Regensburg and Clinic for Neurological Rehabilitation II, Universitätsstr. 84, Regensburg, Germany; 4 Institute of Neuroscience and Medicine (INM) 4, Forschungszentrum Jülich GmbH, Jülich, Germany; 5 Effigos GmbH, Am Deutschen Platz 4, Leipzig, Germany; 6 Synaptic Systems, Göttingen, Germany; University of South Florida, UNITED STATES

## Abstract

Current stroke therapy is focused on recanalizing strategies, but neuroprotective co-treatments are still lacking. Modern concepts of the ischemia-affected neurovascular unit (NVU) and surrounding penumbra emphasize the complexity during the transition from initial damaging to regenerative processes. While early treatment with neurotrophic factors was shown to result in lesion size reduction and blood-brain barrier (BBB) stabilization, cellular consequences from these treatments are poorly understood. This study explored delayed cellular responses not only to ischemic stroke, but also to an early treatment with neurotrophic factors. Rats underwent 60 minutes of focal cerebral ischemia. Fluorescence labeling was applied to sections from brains perfused 7 days after ischemia. Analyses focused on NVU constituents including the vasculature, astrocytes and microglia in the ischemic striatum, the border zone and the contralateral hemisphere. In addition to histochemical signs of BBB breakdown, a strong up-regulation of collagen IV and microglia activation occurred within the ischemic core with simultaneous degradation of astrocytes and their endfeet. Activated astroglia were mainly depicted at the border zone in terms of a glial scar formation. Early treatment with pigment epithelium-derived factor (PEDF) resulted in an attenuation of the usually up-regulated collagen IV-immunoreactivity. However, glial activation was not influenced by treatment with PEDF or the epidermal growth factor (EGF). In conclusion, these data on ischemia-induced cellular reactions within the NVU might help to develop treatments addressing the transition from injury towards regeneration. Thereby, the integrity of the vasculature in close relation to neighboring structures like astrocytes appears as a promising target.

## Introduction

Ischemic stroke represents one of the leading causes of combined death and disability worldwide, associated with long-term socio-economic and personal burden [1, 2]. While intravenous application of recombinant tissue plasminogen activator (rt-PA) within the first hours after symptom onset was demonstrated beneficial in stroke patients [3], limitations had been identified for major vessel occlusion as for instance at the proximal part of the middle cerebral artery [4]. Focusing on this population of stroke patients, characterized by a high risk for malignant edema formation [5], the spectrum of treatment strategies during the early phase of stroke was currently extended by mechanical thrombectomy [6]. However, these treatments are limited to a minority of patients and the development of further neuroprotective co-therapies has halted in the translational process from hopeful basic science into useful clinical applications [7].

During the last decade, the understanding of stroke pathophysiology has substantially improved [8]. The penumbra concept of tissue damage considers time-dependent effects of stroke evolution and describes in terms of a shell-like pattern with an increasing core over time the transition from a temporarily affected, potentially salvageable to an irreversibly impaired brain tissue [9, 10]. As a reflection of such a highly dynamic processes concerning stroke progression and edema formation, a variety of time-critical concepts were conceived, i.e. the biphasic blood-brain barrier (BBB) opening [11, 12], and delayed inflammatory responses [8]. On a cellular level, the perspective of the involved components affected by ischemia has been refined from a neurocentric to a more matured concept, considering the arrangement and interaction of neurons with neighboring glial cells and the endothelium as summarized in the term ‘neurovascular unit’ (NVU) [13–15]. Nevertheless, as therapeutic approaches like those with the free-radical scavenger NXY-059 failed to show any benefit in a clinical setting [16]. Moreover, the application of matrix metalloproteinase inhibitors, assumed to hamper BBB opening with consecutive stroke volume reduction, led to a delayed suppression of neurovascular remodeling with increased ischemic lesions [17]. Therefore, stroke research was assessed with a need for substantial reorientation [18]. Consequently, subsequent efforts are requested to consider both the complexity within the NVU and delayed effects of applied experimental therapies beyond the early phase of stroke [19–22]. With respect to the transition towards irreversibly damaged tissue and the overlapping regenerative processes [23], experimental treatments became attractive that could be administered early after the event with potentially beneficial effects at this stage and also a substantial role for promoting regeneration [24–26].

From a variety of factors that may promote delayed beneficial effects when administered early after the ischemic event, neurotrophic factors became interesting as a class of proteins in the mammalian brain that is critically involved in maintaining neuronal function [27]. For example, preclinical studies have indicated that administration of the brain-derived neurotrophic factor (BDNF) one hour after transient focal cerebral ischemia in the rat caused a marked reduction of stroke volume at 7 days [28]. The pigment epithelium-derived factor (PEDF) as a member of the serine protease inhibitor gene family [29] has shown to activate the microglial metabolism without stimulation and proliferation in cell culture experiments [30]. Further, PEDF was reported to modulate angiogenesis by inhibiting uncontrolled vascular proliferation [31], and has demonstrated anti-inflammatory properties, by decreasing pro-inflammatory factors like the tumor necrosis factor-α as shown in a rat model of retinopathy [32]. With a more focused effect on proliferation, the epidermal growth factor (EGF) as a potential mitogen was discussed to promote neuroprotection [33], whereas experimental treatment with EGF resulted in maintained neuronal density in the hippocampal CA1 region after transient forebrain ischemia in gerbils. In a previous study [34], experimental treatment with PEDF and EGF following 60 minutes of focal cerebral ischemia resulted in an attenuated lesion volume during a 7-days observational period as assessed by serial magnetic resonance imaging (MRI). Moreover, treatment with PEDF led to a significant suppression of the ischemia-related edema formation 48 hours after reperfusion following focal cerebral ischemia. While consequences of experimental treatment with neurotrophic factors following focal cerebral ischemia were reported in terms of a decreased lesion volume and edema formation as well as decreased rates of pro-inflammatory mediators, changes on the cellular level underlying these consequences are poorly understood. However, such information might help to develop more specific treatment strategies considering time-dependent alterations within the ischemia-affected NVU.

The present study investigated histopathological features of NVU constitutes 7 days after transient focal cerebral ischemia, and explored effects of an early experimental treatment with neurotrophic factors. Completing own previous work based on stroke consequences as visualized by MRI in the same ischemia model [34], this study applied multiple fluorescence labeling to allow the detection of concomitantly occurring changes at the vasculature (e.g. collagen IV as basement membrane constituent), microglia and astrocytes including their endfeet.

## Materials and methods

### Study design and content

For the present histochemical study of cellular alterations after experimental treatment with the neurotrophic factors PEDF and EGF, brains were derived from those rats that have been considered in our earlier work on diverse macroscopic characteristics of the ischemic lesion evolution and associated BBB integrity as assessed by serial MRI [34]. In brief, male Sprague-Dawley rats (Janvier, Le Genest-St-Isle, France) with body weights between 250 and 300 g underwent 60 minutes of transient focal cerebral ischemia. The neurobehavioral impact of the ischemic injury was assessed using a 5-point neurologic deficit scale as described earlier [35], and those animals failing to demonstrate the minimally required score of one were excluded from the study. A total of 47 rats have been entered in the study and were allocated to the following experimental groups: Treatment with EGF (n = 16), PEDF (n = 14) and control (saline, n = 17). Details on the surgical procedure for stroke induction and the drug treatment regimens are given below.

Seven days after transient focal cerebral ischemia, animals were deeply anesthetized with pentobarbital (40 mg/kg i.p.) and perfused with ice-cold heparinized phosphate-buffered saline (pH ~7.4). Subsequently, the animals were perfused with freshly prepared 4% paraformaldehyde (PFA) and the brains were carefully removed from the skulls. Brains were then left in 4% PFA for about one week for post-fixation, and subsequently transferred to phosphate-buffered sucrose solution (30%) until histological examinations. During the study process, n = 17 animals were excluded from histological analyses since cerebral hemorrhage had been detected in MRI during the 7-days observational period [34]. Furthermore, n = 4 animals had to be excluded due to substantial tissue damage, impeding sufficient histological analyses. Overall, the present histochemical study is based on n = 26 animals, arranged with n = 10 in the EGF group, n = 6 in the PEDF and n = 10 in the control group. Generally, histochemical analyses including quantifications were done in a blinded fashion to the experimental study group to avoid any bias due to expectations related to the applied experimental treatment with neurotrophic factors.

All animal experiments were performed in accordance with the European Community Council’s directive (86/609/EEC). Experiments complied with the given institutional guidelines for animal care and were done after obtaining approval from the ethics committee for animal laboratories of the Medical Faculty of the University Hospital of Regensburg.

### Experimental focal cerebral ischemia

Transient focal cerebral ischemia was induced by 60 minutes of middle cerebral artery (MCA) occlusion as described previously [12]. In brief, rats were anesthetized using 5% isoflurane (Baxter Deutschland GmbH, Unterschleissheim, Germany) during induction, followed by a maintenance dose of 1.5% isoflurane, applied with a mechanical rodent ventilator (RS Biomed, Sinzing, Germany). The left common carotid artery (CCA) and its bifurcation were exposed carefully following a midline neck incision. To avoid bleeding complications, the occipital artery branches of the external CCA were isolated, ligated, and finally dissected. After careful preparation of the internal carotid artery (ICA), a standardized silicone-coated polyamide 4–0 monofilament (3 cm in length; Ethicon, Johnson & Johnson Medical GmbH, Norderstedt, Germany) was introduced into the ICA and gently advanced forward until a mild resistance was felt, marking the origin of the ipsilateral MCA. The filament was then secured and the neck incision was temporary closed. After 60 minutes, rats were re-anesthetized and the neck incision was re-opened, followed by removal of the monofilament and wound closing. Throughout the surgical procedures, the body temperature was continuously kept at ~ 37.0°C using a commercially available feedback-regulated heating pad (RS Biomed).

### Experimental treatment with neurotrophic factors

Experimental treatment started 3 hours following reperfusion (4 hours after ischemia onset). Applied solutions of EGF and PEDF were prepared to achieve an equimolar dose of brain derived neurotrophic factor (molecular mass 28 kDa, 50 mg/300 g body weight), which previously caused neuroprotective effects after transient focal cerebral ischemia in rats [28]. Dilutions of human recombinant EGF (Biotrend, Cologne, Germany) and PEDF (Creative Biomart, Shirley, NY, USA) were prepared in cold (< 4°C) sterile water for injection to achieve final concentrations of 11.16 mg/1,000 μL as well as 89.28 mg/1,000 μL respectively for a calculated body weight of 300 g. A 10% bolus dose (100 μL) was intravenously applied, followed by the continuous infusion of the remaining volume (900 μL, 225 μL/h for 4 hours), using a micro-infusion pump (Syringe Pump, TSE, Bad Homburg, Germany). Animals in the control group received saline infusions administered in an identical fashion to that of the NTF treatment groups.

### Tissue preparation for multiple fluorescence labeling

Using a freezing microtome, frontal brain sections of 30 μm thickness were cut and collected in 0.1 M Tris-buffered saline (pH 7.4, TBS) containing sodium azide. Prior to all histochemical procedures, free-floating sections were extensively washed with TBS.

The first series of sections was subjected to double fluorescence staining with rabbit-anti-collagen IV and biotinylated *Solanum tuberosum* (potato) lectin (STL). For this purpose, non-specific binding sites of tissues (for subsequently used immunoreagents and lectins) were primarily blocked with 5% normal goat serum (Dianova, Hamburg, Germany, as supplier for Jackson ImmunoResearch, West Grove, PA, USA) in TBS containing 0.3% Triton X-100 for at least 1 hour. Subsequently, the sections were incubated overnight with a mixture of rabbit-anti-collagen IV (Merck Millipore, Billerica, MA; 1:200 in the blocking solution) and biotinylated STL (Vector Laboratories, Burlingame, CA). Sections were then rinsed with TBS and concomitantly reacted with carbocyanine (Cy)3-conjugated donkey-anti-rabbit IgG and Cy2-tagged streptavidin (both from Dianova; 20 μg/mL TBS containing 2% bovine serum albumin = TBS-BSA) for an additional 1 hour.

Consecutive serial sections were simultaneously stained for the astrocytic glial fibrillary acidic protein (GFAP), the ionized calcium binding adapter molecule-1 (Iba) as well as biotinylated STL. In addition, the selected sections were subjected to triple fluorescence labeling of various markers as listed in Table 1. Briefly, the tissue was primarily incubated with different mixtures of primary markers (diluted in TBS containing 5% NDS and 0.3% Triton X-100) for 16–20 hours, followed by incubation with a mixture of fluorochromated secondary immunoreagents for 1 hour. All fluorescent antibodies and streptavidin conjugates were obtained from Dianova and used at 20 μg/mL TBS-BSA.

**Table 1:** Triple fluorescence labeling.

First marker Primary antibodies/lectin	First marker Secondary reagents*	Second marker Primary antibodies/lectin	Second marker Secondary reagents*	Third marker Primary antibodies/lectin	Third marker Secondary reagents*
biotinylated STL (20 μg/ml; Vector, Burlingame, CA, SA)	Cy2-streptavidin	rabbit-anti-albumin (1:200; Synaptic Systems, Göttingen, Germany)	Cy3-donkey-anti- rabbit IgG		
guinea pig-anti-GFAP (1:200; Synaptic Systems)	Cy2-donkey-anti- guinea pig IgG	rabbit-anti-Iba (1:200; Synaptic Systems)	Cy3-donkey-anti- rabbit IgG	biotinylated STL (20 μg/ml; Vector)	Cy5-streptavidin
biotinylated STL (20 μg/ml; Vector)	Cy2-streptavidin	rabbit-anti-collagen IV (1:200; Merck Millipore, Billerica, MA, USA)	Cy3-donkey-anti- rabbit IgG		
goat-anti-collagen IV (1:100; Merck Millipore)	Cy3-donkey-anti-goat IgG	rabbit-anti-fibronectin (1:400; (AbD Serotec, Oxford, UK)	Cy2-donkey-anti- rabbit IgG	biotinylated STL (20 μg/ml; Vector)	Cy5-streptavidin
goat-anti-collagen IV (1:100; Merck Millipore)	Cy3-donkey-anti-goat IgG	rabbit-anti-aquaporin 4 (1:200; Alomone, Jerusalem, Israel)	Cy2-donkey-anti- rabbit IgG	biotinylated STL (20 μg/ml; Vector)	Cy5-streptavidin
rabbit-anti-aquaporin 4 (1:200; Merck Millipore)	Cy3-donkey-anti-rabbit IgG	guinea pig-anti-GFAP (1:200; Synaptic Systems)	Cy2-donkey-anti- guinea pig IgG	biotinylated STL (20 μg/ml; Vector)	Cy5-streptavidin
rabbit-anti-S100β (1:600; Synaptic Systems)	Cy3-donkey-anti- rabbit IgG	guinea pig-anti-GFAP (1:200; Synaptic Systems)	Cy5-donkey-anti- guinea pig IgG	biotinylated STL (20 μg/ml; Vector)	Cy2-streptavidin
rabbit-anti-VEGF (1:400; Thermo Scientific, Fremont, CA, USA)	Cy3-donkey-anti- rabbit IgG	guinea pig-anti-GFAP (1:200; Synaptic Systems)	Cy2-donkey-anti- guinea pig IgG	biotinylated STL (20 μg/ml; Vector)	Cy5-streptavidin
mouse-anti-CD68 (1:50; AbD Serotec)	Cy3-donkey-anti- mouse IgG	rabbit-anti-Iba (1:200; Synaptic Systems)	Cy2-donkey-anti- rabbit IgG	guinea pig-anti-GFAP (1:200; Synaptic Systems)	Cy5-donkey-anti- guinea pig IgG

*All fluorescent immunoreagents were obtained from Dianova (Hamburg, Germany) as supplier for Jackson ImmunoResearch (West Grove, PA, USA). Abbreviations: STL–*Solanum tuberosum* agglutinin (= potato lectin); Iba–ionized calcium binding adapter molecule-1; GFAP–glial fibrillary acidic protein; VEGF–vascular endothelial growth factor

In control experiments, antibodies were omitted, resulting in the absence of cellular signals. Finally, all sections were extensively rinsed with TBS, quickly washed with distilled water, mounted on to the slides, air-dried and coverslipped with Entellan in toluene (Merck, Darmstadt, Germany).

### Quantification of collagen IV-, GFAP- and Iba-immunoreactivity

A first screening of immunolabeled structures and estimation of ischemia-affected regions was performed with an Axioplan fluorescence microscope (Zeiss, Oberkochen, Germany).

For quantification of immunosignals for collagen IV, GFAP and Iba, 3 consecutive brain sections were selected while the middle section had to present the visually detected maximum of ischemic tissue affection, added by a rostral and caudal section at a distance of 300 μm, respectively. Using a Keyence fluorescence microscope (BZ-9000 series; Keyence, Osaka, Japan), 3 regions of interest (ROIs) were manually inserted in each section, capturing the striatal ischemic area (assumed to represent the ischemic core), the area of attenuated tissue affection in the medial striatum (assumed to represent the ischemic border zone) as well as the striatum of the contralateral hemisphere (used as control). Applying a 40x magnification (0,95/0.14 mm, aperture 1.0; settings: exposure time 1/10 sec, gain 0), these ROIs were used to capture the red fluorescence signal assigned to demonstrate collagen IV and Iba as well as the green fluorescence signal for GFAP. Overall, 3 figures were made per section, resulting in a total of 9 images per animal. These images were further processed with ImageJ (open source, National Institutes of Health, Bethesda, MD, USA) to calculate the staining density and intensity of the applied markers, visualized by immunofluorescence labeling. To consider the slightly different brightness among the applied labeling, the following thresholds for the detection of positive pixels were used: 22 counts for collagen IV, 23 counts for GFAP, and 24 counts for Iba. Pixels achieving these thresholds entered the subsequent calculations. Thereby, staining density complied with number of positive pixels within the ROI that contains a theoretical maximum of 151,111 pixels, while staining intensity complied with the degree of luminosity as scaled from 0.00 to 99.99. Finally, mean values were built between the 3 sections analyzed per animal, resulting in a single value for each of the 3 ROIs (core, border zone and control).

For illustration, high-resolution images were taken from selected sections using the confocal laser-scanning microscope 510 Meta (Zeiss, Jena, Germany) as well as a Keyence fluorescence microscope (BZ-9000 series; Keyence). Original images were processed with PowerPoint for Mac 2011 (Microsoft, Redmond, WA, USA). If indicated, brightness, contrast or intensity were slightly adjusted without creating or deleting signals.

### Statistical analyses

Calculations were performed with the SPSS version 20.0 (IBM Corp., New York, NY, USA). In addition to descriptive statistics, the Wilcoxon test and the Kruskal-Wallis test (added by an exact test with a preset of 5 minutes) were used to check for statistical significance between the groups. Further, Pearson correlations were applied to explore interrelations between different parameters. Generally, a p-value < 0.05 was considered as statistically significant. The underlying data set and used syntax for calculations are available as S1 and S2 Files.

## Results

Histochemical analyses were performed with brain tissues from n = 26 rats that underwent 60 minutes of transient focal cerebral ischemia with an additional observation period of 7 days, corresponding with the last *in vivo* (MRI) data reported in our previous work [34].

### Delayed vascular and glial changes related to experimental focal cerebral ischemia

**Fig 1:**
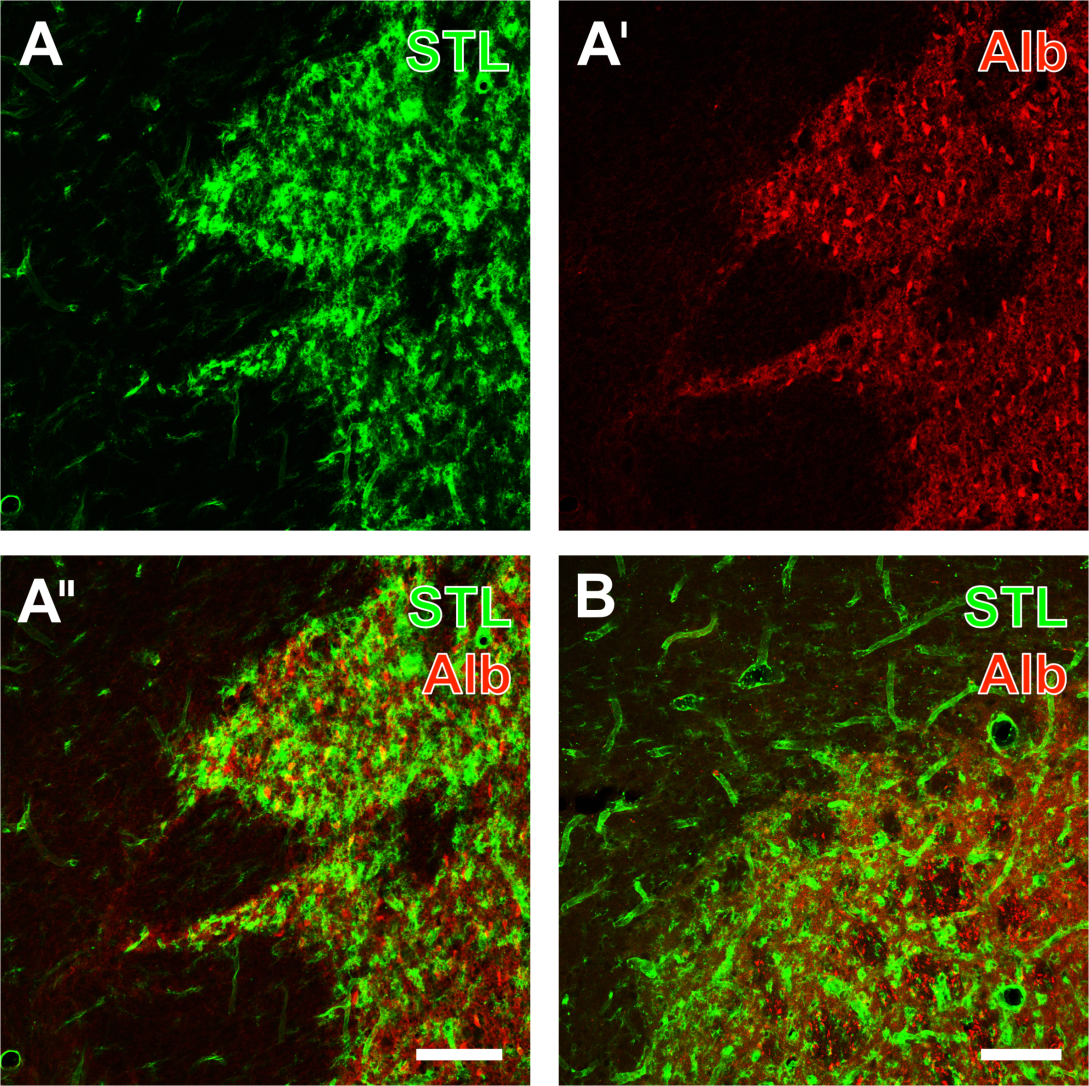
Combined staining of endothelial cells and albumin-immunoreactivity indicating significant alterations blood-brain barrier (BBB) integrity 7 days after transient focal cerebral ischemia in the neocortex and the striatal border region. Lectin-histochemical staining with biotinylated *Solanum tuberosum* lectin (STL) and Cy2-streptavidin (A) revealed a persisting, but thinned endothelium in stroke-affected neocortical areas. The concomitant Cy3-immunolabeling based on rabbit-anti-serum albumin (Alb, A’) showed the BBB permeability marker within the parenchyma which became even clearer by the merged picture (A”). The overlay of STL and albumin clearly revealed stroke-induced leakage of the BBB resulting in intra-parenchymal albumin-immunoreactivity also visible in several perikarya, whereas another animal displayed less albumin-immunopositive somata in an infarcted striatal border region (B). Scale bar in A” (also valid for A and A’) = 75 μm, in B = 100 μm.

To obtain an overview about the vasculature and associated BBB integrity at one week following transient MCA occlusion without experimental treatment, a concomitant staining of endothelial cells and endogenous albumin-immunoreactivity was performed. Thereby, endothelial staining with the biotinylated STL and Cy2-streptavidin resulted in persisting, but partially thinned endothelia in ischemic neocortical areas (Fig 1A) as well as in the striatum (Fig 1B) as the primarily stroke-affected region due to the employed filament-based model of transient MCA occlusion. Concomitant Cy3-immunolabeling based on rabbit-anti-serum albumin showed the leakage of endogenous albumin into the parenchyma, clearly visible in the overlays of staining patterns (Fig 1A” and 1B”), which indicated a substantially altered integrity of the blood-brain barrier in ischemic areas.

**Fig 2:**
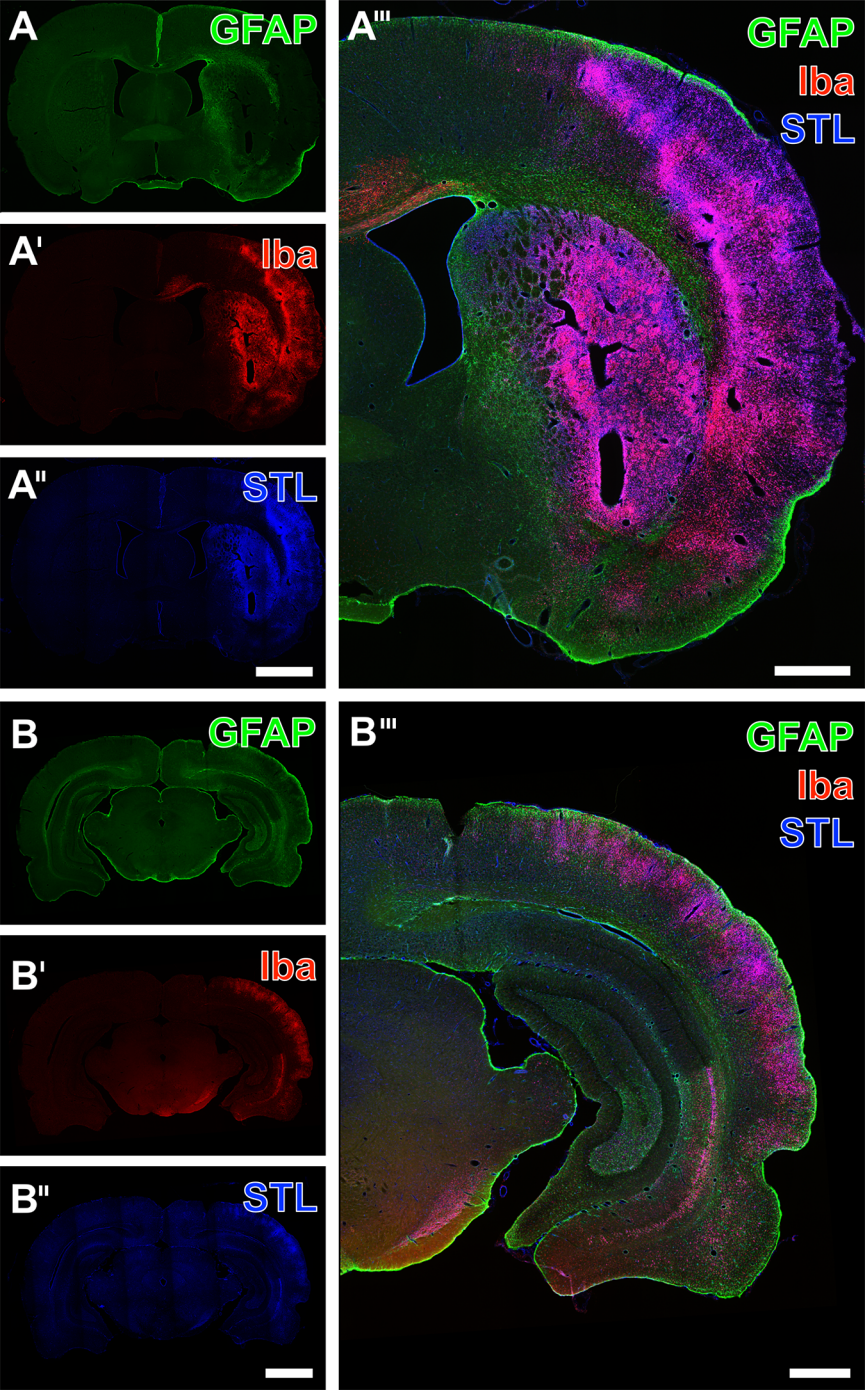
Exemplified overview scans of carbocyanine triple fluorescence labeling of GFAP in astroglia, Iba as marker for microglia/macrophages and STL-binding sites of endothelial cells as well as macrophages 7 days after transient focal cerebral ischemia in non-treated rats. GFAP-immunoreactivity appeared decreased in areas of ischemia-related tissue damage of both the striatum and the neocortex (A, B). On the contrary, enhanced Iba-immunoreactivity (A’, B’) and STL-staining (A”, B”) were found in the ischemic areas. Merge of the staining patterns impressively visualized concomitant cellular alterations in the ischemia-affected striatum and neocortex. Scale bar in A” (also valid for A and A’) = 250 μm, in B” (also valid for B and B’) = 250, in A”‘ and B”‘ = 100 μm.

**Fig 3:**
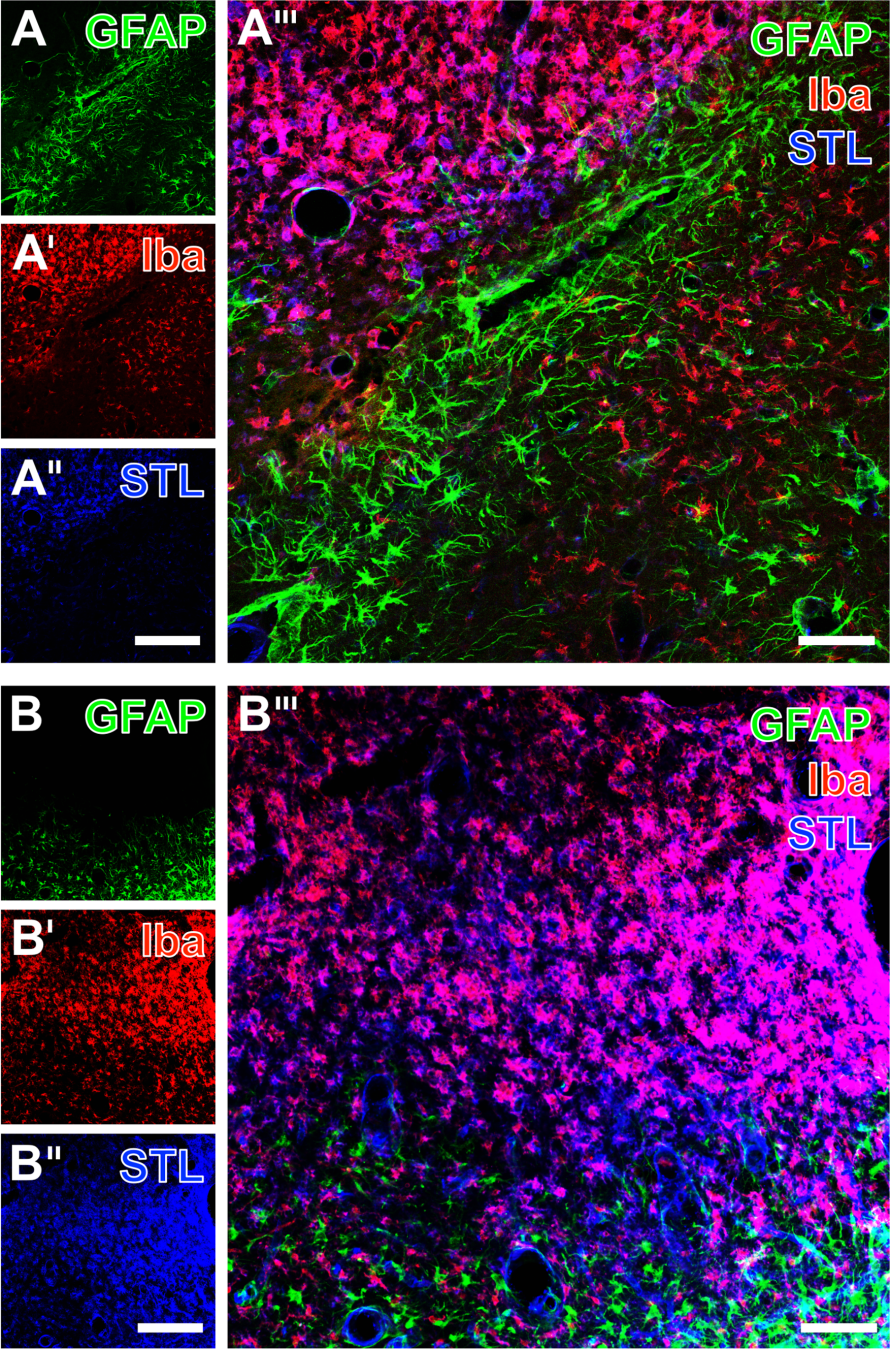
Triple fluorescence-based staining of GFAP (astroglia), Iba (microglia/macrophages) and STL-binding (endothelial cells as well as macrophages) at the ischemia-affected striatum (A-A”‘) and neocortex (B-B”‘) of non-treated animals. Thereby, Cy2-immunolabeling of GFAP demonstrated a glial scar formation composed of activated astrocytes (A) which was nearly devoid of Cy3-immunosignals for Iba (A’) and separates ramified microglia in the apparently less affected tissue from numerous ameboid cells in the ischemic region, which also showed a strong Cy5-staining of STL (color-coded in blue; A”). Merging the staining patterns clearly demonstrated the allocation of Iba-immunoreactivity and STL-binding sites in purple-appearing cells (A”‘). Neocortical Cy2-immunolabeling of GFAP revealed a pattern of apparently activated astrocytes (B), and in upper layers numerous ameboid microglia/macrophages, revealed by Cy3-immunodecoration of Iba (B’) and Cy5-staining of STL (color-coded in blue; B”)–appearing purple in the overlay (B”‘). Scale bars: in A” and B” (also valid for A, A’, B and B’) = 200 μm, in A”‘ and B”‘ 75 = μm.

To address glial changes related to experimental focal cerebral ischemia, triple immunofluorescence labeling including astroglial GFAP, Iba in microglia/macrophages as well as STL-binding sites of endothelial cells/macrophages was performed (Fig 2). Overview scans thereby indicated drastic cellular reactions, while GFAP-immunolabeling was found to decrease in the area of maximum tissue damage (Fig 2A and 2B). In contrast, Iba-immunoreactivity and STL-staining appeared strongly up-regulated in areas of ischemic affection (Fig 2A’ and 2A” as well as 2B’ and 2B”). The respective merged figures clearly indicated concomitant changes of the addressed cellular markers (Fig 2A”‘ and 2B”‘). When focusing on the striatum (Fig 3A–3A”‘) and the neocortex (Fig 3B–3B”‘) in non-treated rats, GFAP-immunolabeling revealed a scar formation of morphologically altered (most likely activated) astroglia in close vicinity to the ischemia-affected area in the striatum (Fig 3A) and–to a much lesser degree–in the neocortex (Fig 3B). Simultaneously, Iba visualized ramified microglia in obviously hardly affected tissue (Fig 3A’) as well as numerous ameboid cells in the ischemic regions (Fig 3B’). Based on the presence of numerous reactive immune cells in the ischemic regions, STL also revealed ameboid cells in addition to the endothelium (Fig 3A” and 3B”). The overlay of staining patterns clearly elucidated the allocation of Iba-immunoreactivity and STL-binding sites in many immune cells, predominantly located in the severely affected region (upper part of both figures), whereas the astroglial reaction as visualized by GFAP was mainly found in terms of a bordering rim (Fig 3A”‘ and 3B”‘).

**Fig 4:**
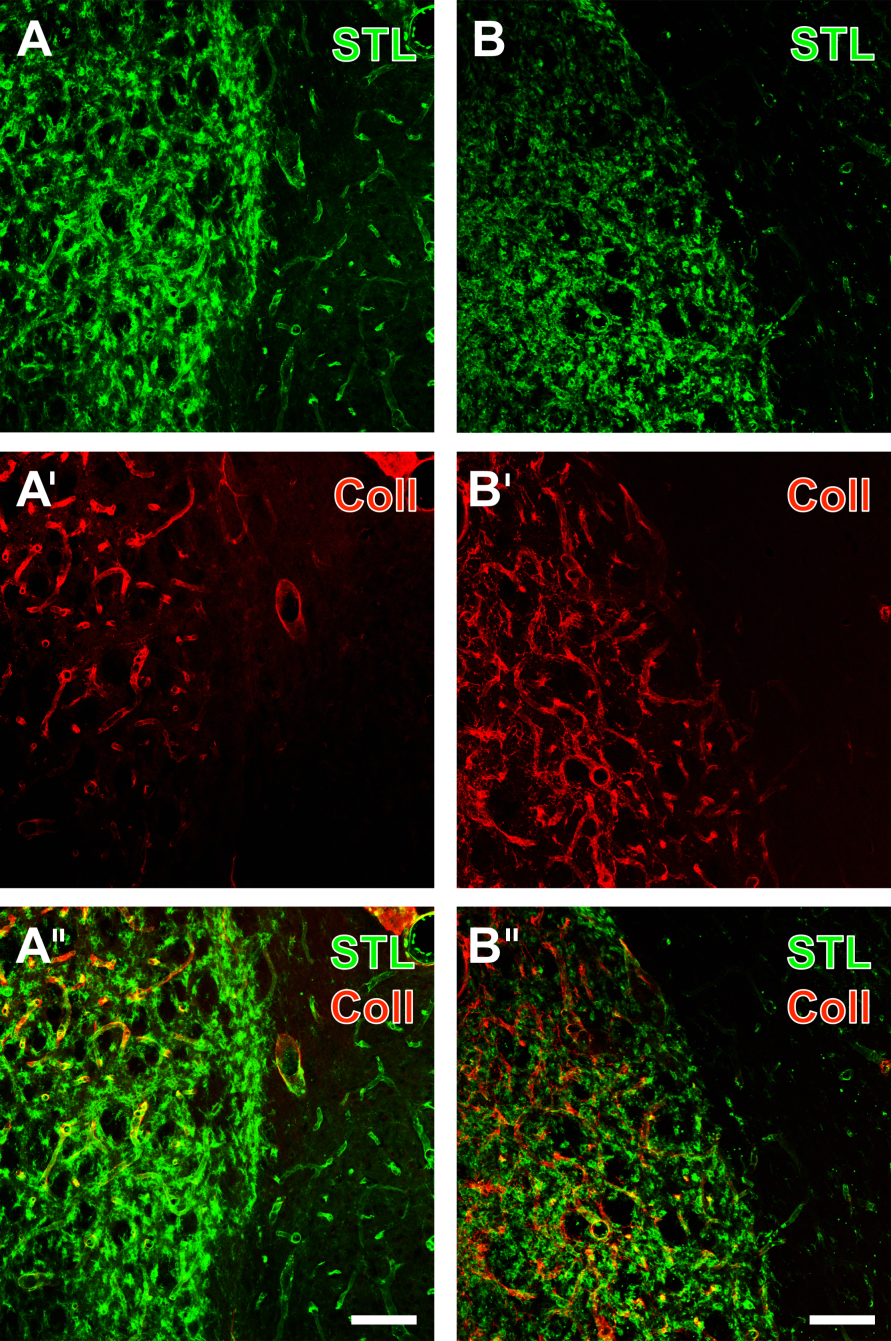
**Fluorescence-based staining of binding sites for STL in endothelial cells and activated microglia/macrophages (Cy2, green) combined with Cy3-immunolabeling of collagen IV (Coll, red) in the basal membranes 7 days after transient focal cerebral ischemia in the border region between neocortex and infarcted striatum from an EGF-treated animal (A-A”) and from a non-treated rat (B-B”).** Selectively STL-marked striatal endothelial cells (A) were found to appear sharply separated from the ischemia-affected region, which was predominantly filled by activated microglia/macrophages. Additionally stained vessels were frequently and strongly immunolabeled for collagen IV (A’), while the allocation of both markers became clearly visible in the merged figure (A”). Similarly, STL-staining in tissues from the non-treated group clearly delineated the ischemia-affected lateral striatum, containing many activated microglia/macrophages and the neocortex devoid of such cells, but displayed weaker stained endothelial cells (B). Concomitantly revealed collagen IV-immunoreactivity was strongly up-regulated solely in the ischemic striatum (B’) in close vicinity to immune cells (B”). Scale bar in A” and B” (also valid for all other micrographs) = 100 μm.

In order to investigate the constitutes of the vasculature under ischemic conditions in more detail, the visualization of STL-binding sites in endothelial cells and activated microglia/macrophages was combined with Cy3-immunolabeling of collagen IV, typically located in basal membranes (Fig 4). This double staining revealed a homogeneous pattern of striatal endothelial cells and mostly activated microglia/macrophages (Fig 4A and 4B) in the border zone between the ischemia-affected striatum and only partially involved neocortex, a feature that was present in both non-treated (Fig 3B–3B”) and EGF-treated animals (Fig 4A–4A”). Remarkably, severe ischemia led to a strong up-regulation of collagen IV-immunoreactivity (Fig 4A’ and 4B’), while the allocation with STL clearly demonstrated the considerable overlap of both vascular markers in the merged figures (Fig 4A” and 4B”).

### Vascular and glial alterations in relation to the ischemic lesion in untreated animals

**Fig 5:**
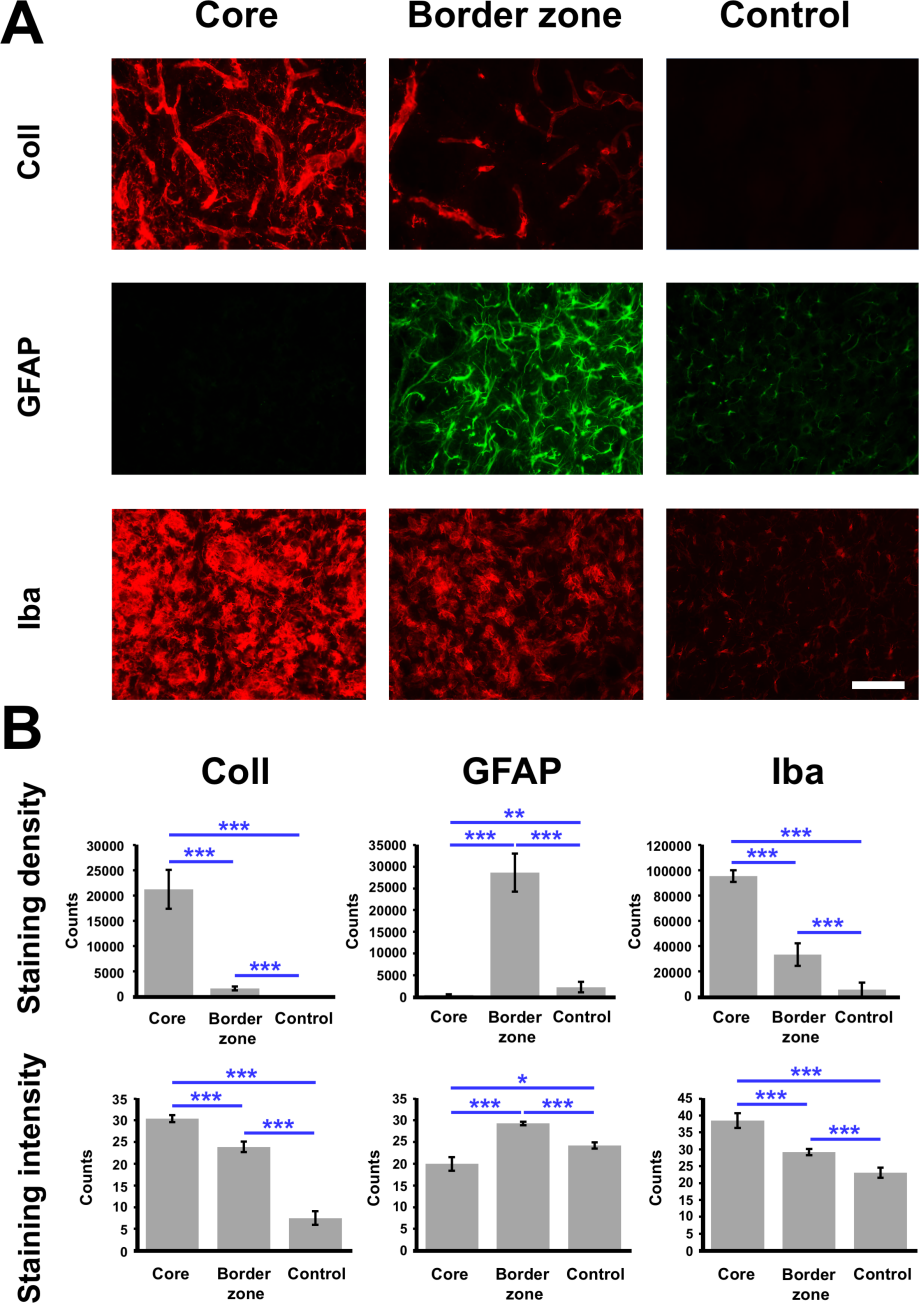
**Exemplified fluorescence staining of collagen IV (Coll), GFAP and Iba depending on the region with respect to the primary ischemic lesion, i.e. the ischemic core, the ischemic border zone and a control area located at the contralateral, non-affected hemisphere (A) and respective quantification in the overall sample (B).** A strong immunolabeling was found for collagen IV and Iba at the ischemic core, diminishing towards the border zone and the control region, while most impressive GFAP-immunostaining was seen at the ischemic border zone in terms of a glial scar formation. Quantitative analyses confirmed the qualitative findings in terms of a most prominent up-regulation of immunoreactivities for collagen IV and Iba in the ischemic core, while the strongest immunoreactivity for GFAP was seen at the ischemic border zone. Scale bar: in A (also valid for all other micrographs) = 75 μm. Bars represent means and added lines represent the standard error of means. *, p < 0.05; **, p < 0.01; ***, p < 0.001.

Three predefined regions (ischemic core, ischemic border zone and a contralateral control area) were investigated concerning immunoreactivities for collagen IV as a vascular marker as well as GFAP and Iba as markers for two distinct glial populations. Applying these 3 regions, on first glance collagen IV-immunoreactivity was found to decrease gradually from the ischemic core towards the ischemic border zone with a nearly complete loss in the control area (Fig 5A). In a quite opposite pattern, GFAP presented a nearly abolished immunoreactivity in the ischemic core and a strong up-regulation in the ischemic border zone, while only a weak signal was seen in the striatum of the contralateral hemisphere. Concomitantly, Iba-immunolabeling was strongly increased in the ischemic core with a diminishing course towards the ischemic border zone and the control region.

Subsequent quantitative analyses in the overall sample confirmed the observed patterns for staining density and intensity (Fig 5B). In detail, a highly significant decrease of collagen IV- and Iba-staining was found at the ischemic border zone with respect to the ischemic core (p < 0.001 for both markers; Wilcoxon test), while the signals were further reduced in the contralateral striatum (p < 0.001 for both markers; Wilcoxon test). On the contrary, staining density and intensity for GFAP increased significantly between the ischemic core and the ischemic border zone (p < 0.001; Wilcoxon test), and decreased towards the contralateral hemisphere (p < 0.001; Wilcoxon test).

### Vascular and glial alterations after experimental treatment with PEDF and EGF

**Fig 6:**
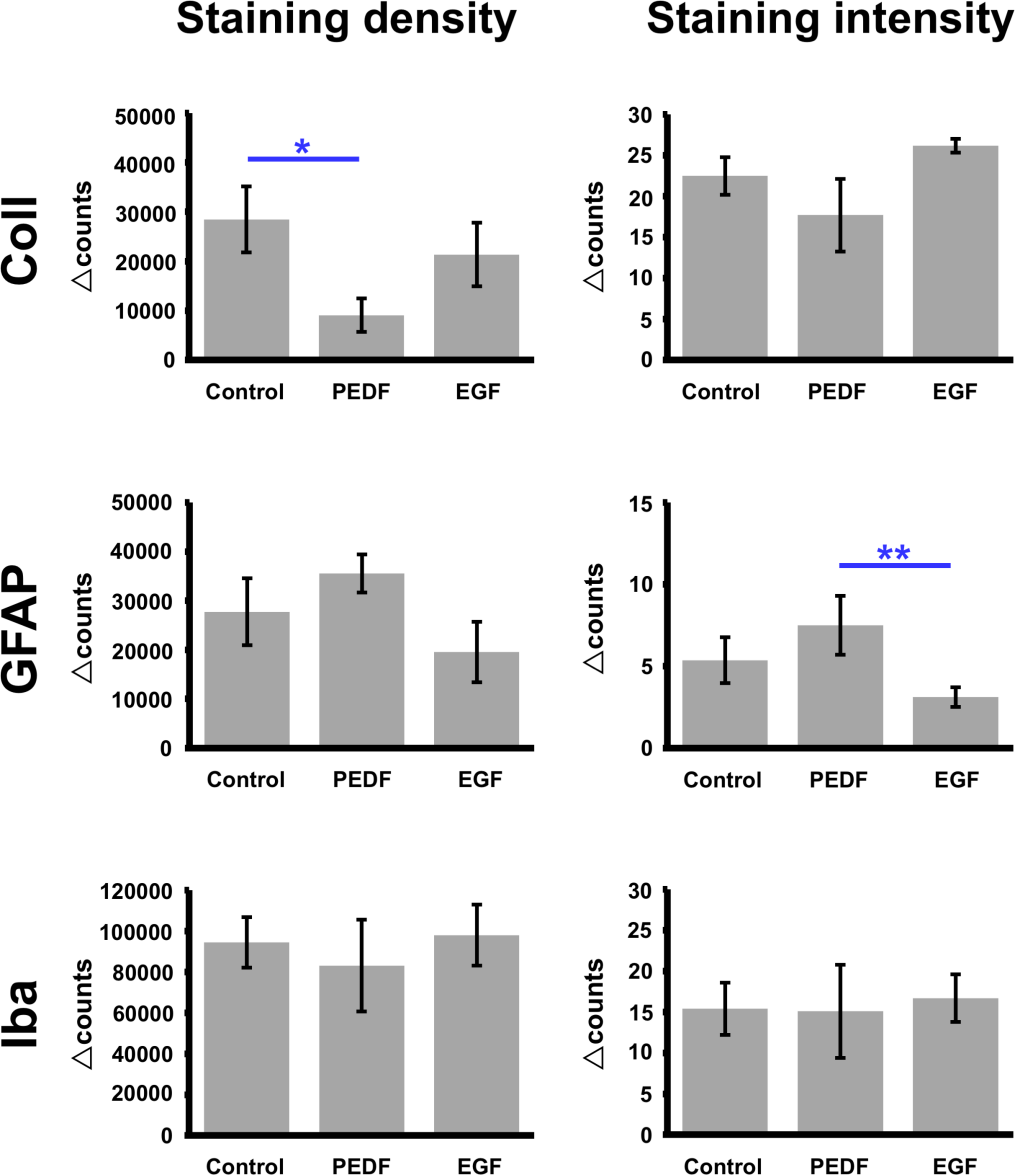
Quantitative analyses of immunoreactivities for collagen IV (Coll), GFAP and Iba in terms of inter-hemispheric differences (Δ-vales) with respect to the contralateral, non-affected side, depending on experimental treatment with neurotrophic factors. Treatment with PEDF was found to significantly attenuate the usually detected up-regulation of collagen IV in ischemia-affected subcortical regions, whereas EGF did not influence collagen IV expression as compared to the control group. However, experimental treatment with PEDF and EGF did not alter immunosignals for GFAP and Iba significantly as compared with the control group. When focusing on potential effects between both experimental treatment, EGF resulted in an attenuation of the usually up-regulated GFAP-immunoreactivity when compared to the treatment with PEDF. Bars represent means and added lines represent the standard error of means. *, p < 0.05; **, p < 0.01.

Based on the observed histochemical changes that were most prominent comparing the ischemic core and the contralateral hemisphere for collagen IV and Iba as well as between the ischemic border zone and the contralateral hemisphere for GFAP, these differences were used for further analyses on the impact of experimental treatment with EGF and PEDF (Fig 6). As compared to the control group, PEDF was found to attenuate the usually observed up-regulated collagen IV-immunoreactivity after ischemia, while statistical significance was only obtained for staining density (p = 0.016; Kruskal-Wallis-test). However, experimental treatment with EGF did not result in a markedly changed density and intensity of collagen IV-staining.

Concerning the applied glial markers GFAP and Iba, treatment with PEDF tended to a slightly intensified staining density and intensity for GFAP, which, however, failed statistical significance (p = 0.220, p = 0.492). Treatment with EGF did not result in a markedly different histochemical pattern of GFAP when compared with the control group. However, an attenuation of the ischemia-caused up-regulated intensity of GFAP-immunolabeling in the ischemic border zone was found after treatment with EGF when compared with the treatment with PEDF (p = 0.003; Kruskal-Wallis-test). Unexpectedly, staining density and intensity for Iba were not altered by experimental treatment with PEDF or EGF.

### Interrelation between vascular and glial alterations depending on experimental treatment

**Fig 7:**
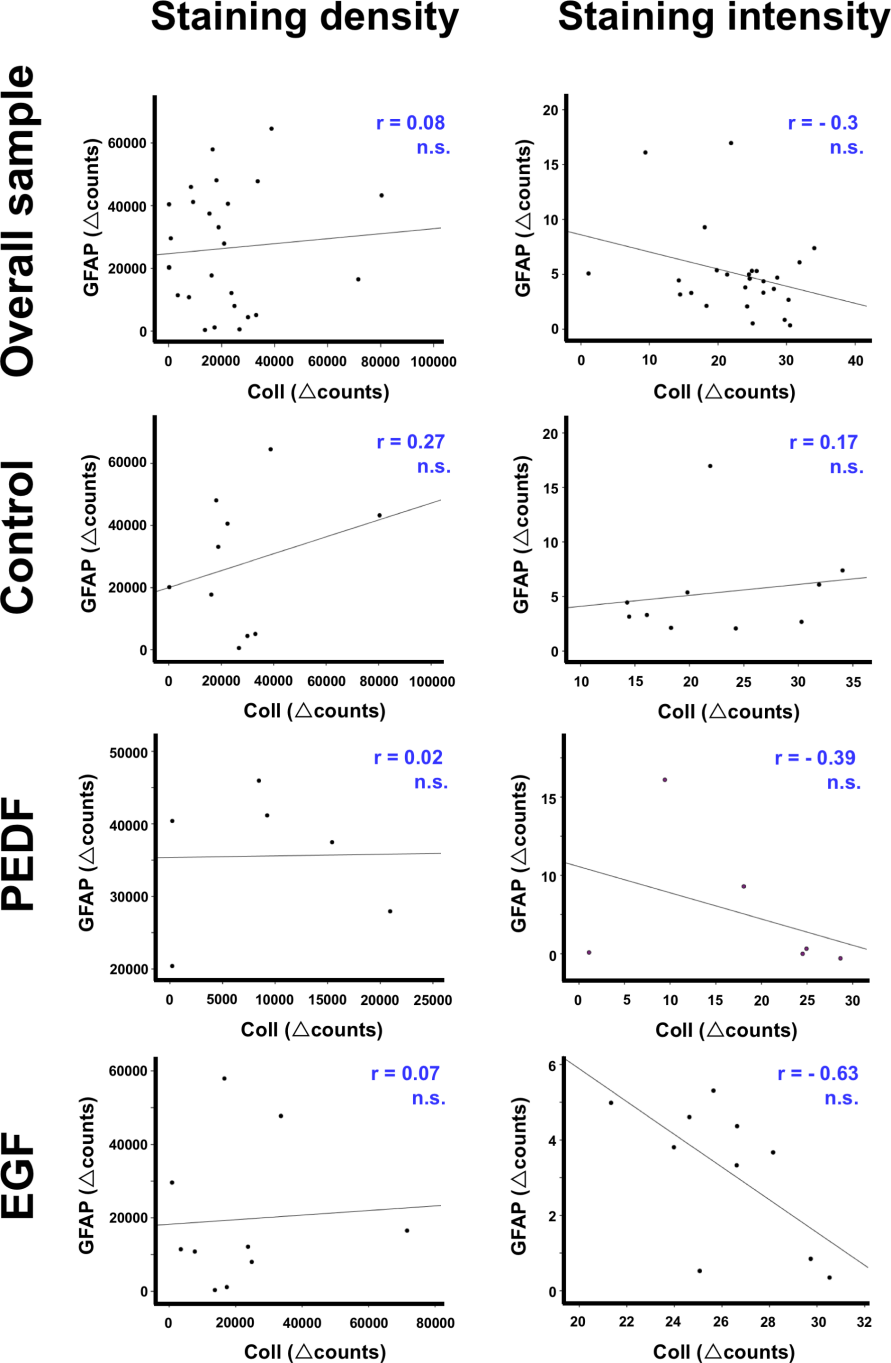
Correlation plots visualizing Δ-values for collagen IV and GFAP in the overall sample and with respect to experimental treatment with neurotrophic factors. r, Pearson correlation coefficient; n.s., non significant.

Additional analyses focused on the statistical interrelation between histochemical alterations of collagen IV as a vascular element and astroglial GFAP (Fig 7). In the overall sample including the control group, correlation coefficients were nearly zero and p-values far off a statistical significance (ranging between 0.142 and 0.692) indicated a lacking statistical interrelation between the addressed vascular and glial component. While focusing on the groups treated with PEDG or EGF, comparable coefficients were found (p-values ranging between 0.441 and 0.972), except the staining intensity after treatment with EGF, demonstrating a relatively strong negative coefficient (r = -0.63), barely missing statistical significance (p = 0.051), indicating–at least in terms of a trend–an opposite reaction of GFAP and collagen IV.

### Additional cellular alterations within the ischemia-affected neurovascular unit

In subsequent qualitative analyses, additional astroglial markers and the interface to the extracellular matrix were investigated with respect to the vasculature in diverse ischemic regions.

**Fig 8:**
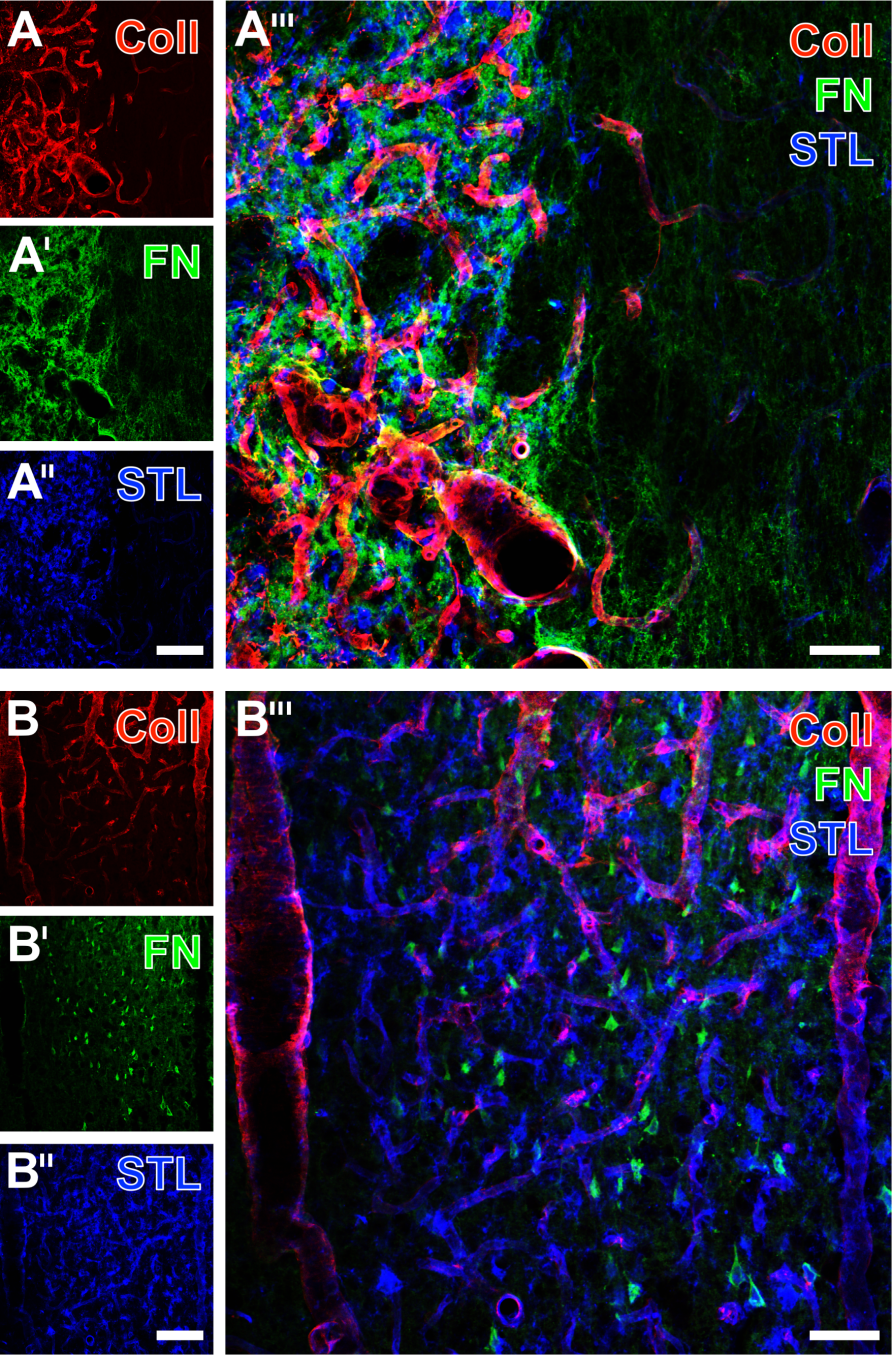
**Triple fluorescence staining of fibronectin (FN), collagen IV (Coll) and STL in the striatum (A-A”‘) and at the neocortex (B-B”‘) of non-treated rats 7 days after transient focal cerebral ischemia.** Dense Cy2-immunolabeling of FN (A’) was seen in the ischemia-affected region simultaneously showing strongly up-regulated collagen IV-immunoreactivity (A) intermingled by lectin-histochemically stained microglia/macrophages (A”, STL color-coded in blue). Concomitantly, STL revealed some apparently healthy endothelial cells in the surrounding tissue. Merging the staining patterns (A”‘) revealed that the region with up-regulated collagen IV was somewhat more extended than the regions with enhanced signals for fibronectin and microglia/macrophages. Fibronectin staining in the neocortex (B’) elucidated this protein not only in the neuropil, but also in numerous somata within the ischemia-affected tissue, which displays only partially up-regulated collagen IV (B) and persisting STL-stained vessels (B”). Merging the staining patterns (B”‘) showed some fibronectin-containing cells with STL-labeling. Scale bars: in A” and B” (also valid for A, A’, B and B’) = 100 μm, in A”‘ and B”‘ = 50 μm.

Thereby, triple fluorescence labeling demonstrated the concomitant up-regulation of the extracellular matrix protein fibronectin and collagen IV, combined with STL staining in the ischemia-affected striatum (Fig 8A–8A”‘) and neocortex (Fig 8B–8B”‘) without experimental treatment. The affected tissue was characterized by dense immunolabeling of fibronectin often predominantly in the neuropil (Fig 8A), but also in somata (Fig 8B). In the same regions, vascular-associated collagen IV appeared strongly up-regulated (Fig 8A’ and 8B’) and allocated with the STL-stained endothelium and microglia/macrophages (Fig 8A” and 8B”). While in the presented striatal region, STL-marked endothelial cells remained restricted to tissue devoid of visualized immune cells (Fig 8A”), both cell types were intermingled in the exhibited cortical areas (Fig 8B”). The overlay of staining patterns (Fig 8A”‘ and 8B”‘) elucidated that the regions with enhanced fibronectin were somewhat larger than those with up-regulated collagen IV-staining.

**Fig 9:**
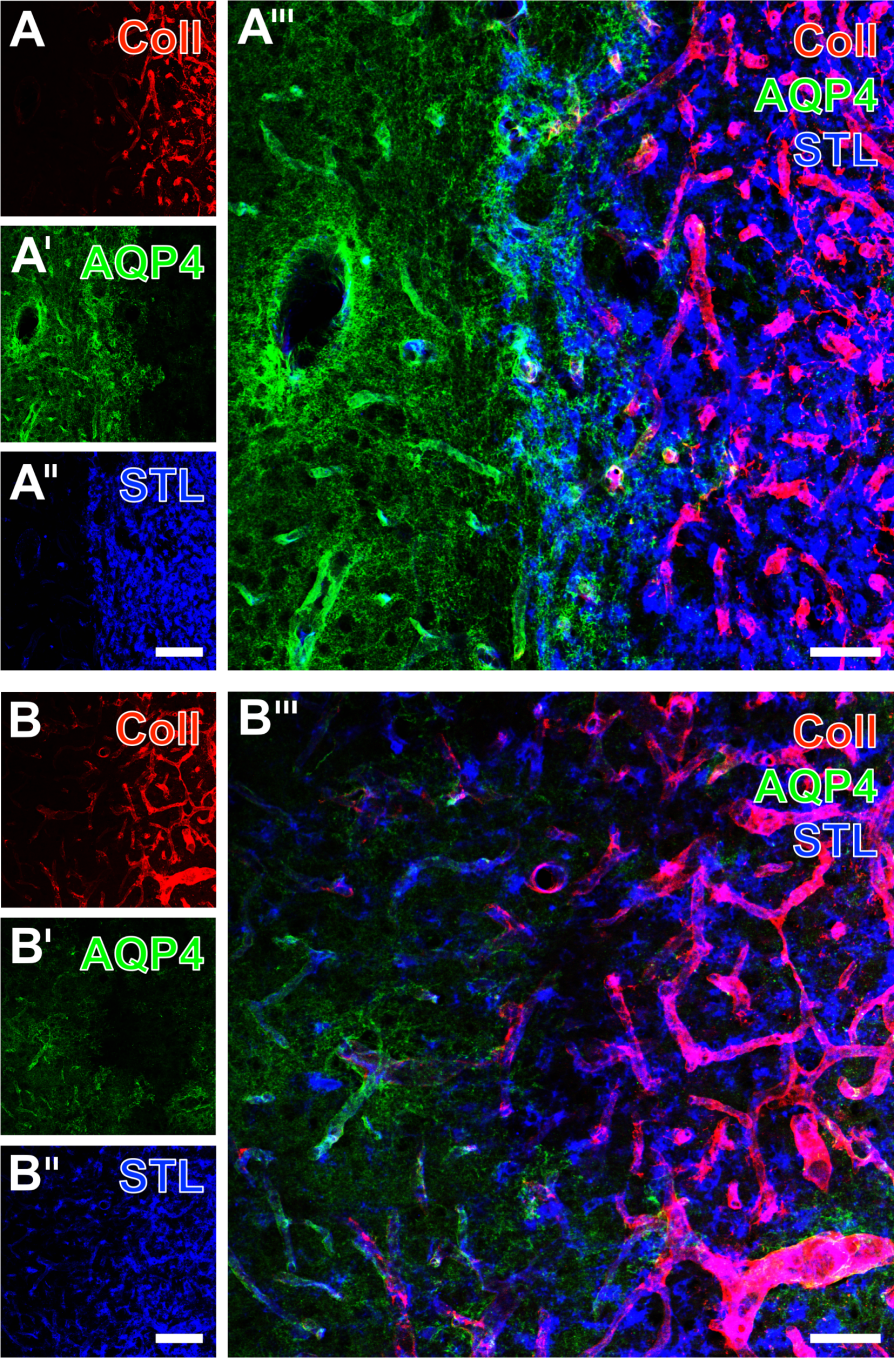
**Concomitant Cy-staining of collagen IV (Coll), aquaporin-4 (AQP4) and STL-binding sites in the lateral striatal border (A-A”‘) and in the neocortex (B-B”‘), exemplarily shown in non-treated rats 7 days after transient focal cerebral ischemia.** The strongly up-regulated collagen IV (A) was absent in the complementary aquaporin-4-stained region (A’), displaying both immunoreactive vessels and neuropil. In parallel, STL-staining (A”) showed some faintly appearing vessels in the aquaporin-4-immunopositive region and densely populated microglia/macrophages, mostly allocated with up-regulated vascular collagen IV. The overlay of the staining patterns (A”‘) revealed a turquoise rim with preserved aquaporin-4-immunoreactivity and STL-positive immune cells, but devoid of collagen IV-immunolabeling. In a representative neocortical ischemic border zone (B), aquaporin-4-immunostaining was mostly found in vessels and the neuropil of hardly affected tissue. However, some vessels with allocated aquaporin-4 and collagen IV–as signs for ischemic tissue–were observed (lower part in B, B’). Concomitantly up-regulated collagen IV (B’) and the predominantly vascular-associated STL-staining (B”) appeared not completely in a complementary manner, which became even clearer after merging the staining patterns (B”‘). Scale bars: in A” and B” (also valid for A, A, B and B’) = 100 μm, in A”‘ and B”‘ = 50 μm.

To address astrocyte endfeet as the interfering structure between the astroglial soma and the vasculature, the detection of collagen IV and STL-binding sites was combined with the immunolabeling of aquaporin 4 in the ischemia-affected striatum (Fig 9A–9A”‘) and neocortex (Fig 9B–9B”‘) originating from control animals. Here, in ischemic regions the up-regulation of collagen IV (Fig 9A and 9B) is accompanied even by a nearly absent immunostaining for aquaporin-4 (right part of Fig 9A’), or a weak co-localization in terms of a preserved vessel-associated aquaporin-4 (lower right part in Fig 8B’). Additional STL-staining revealed in the striatum some endothelial cells in the aquaporin-4-immunopositive region and numerous microglia/macrophages in the infarcted tissue (Fig 9A”), whereas the presented neocortical area also shows intermingled endothelial and immune cells (Fig 9B”). The overlay of striatal staining patterns demonstrated a rim-like pattern of preserved aquaporin-4-immunoreactivity, partially overlapping with STL-positive immune cells, but devoid of detectable collagen IV (Fig 9A”‘). On the other hand, the merge of neocortical staining patterns clarified the occurrence of up-regulated collagen IV also in regions with predominantly vascular STL and weak aquaporin-4 staining (Fig 9B”‘).

**Fig 10:**
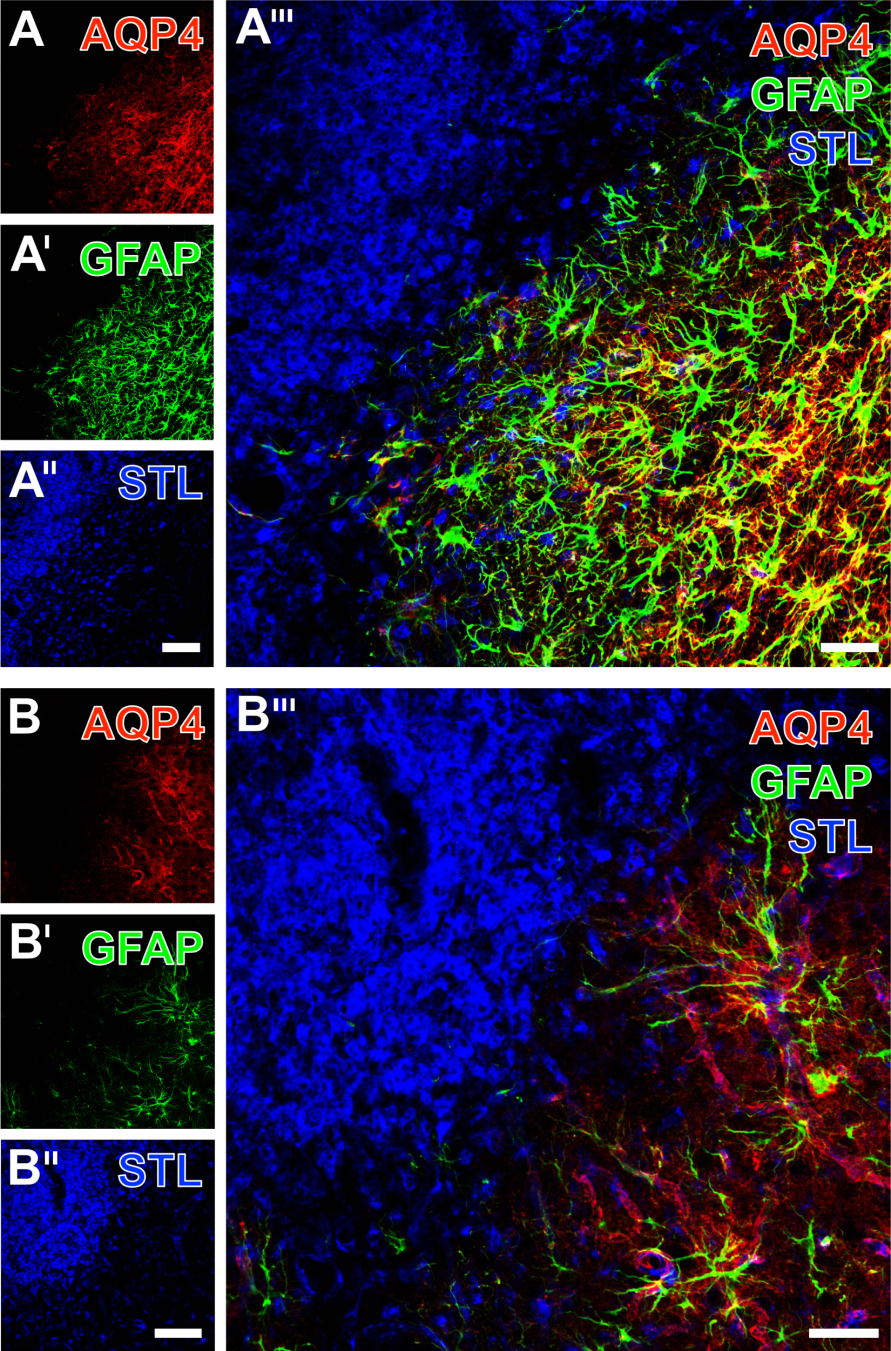
Simultaneous detection of aquaporin-4 (AQP4) on astrocytic endfeet, glial fibrillary acidic protein (GFAP) revealing a large portion of astroglia and STL in endothelial cells and microglia/macrophages at the ischemic striatum of non-treated rats. Aquaporin-4-immunostaining (A) in both the vessels and neuropil remained absent at the infarcted tissue and became only visible in the surrounding, apparently less affected tissue, which displayed numerous activated astrocytes as indicated by a strong GFAP-immunoreactivity (A’). This region also contained some vessels stained by STL (A”), but this marker predominantly revealed densely packed microglia/macrophages in the ischemic region. Merging these staining patterns elucidated the complementary occurrence of immune cells and both astroglial markers. At higher magnification (B), the sharp border between a zone with vascular aquaporin-4-immunoreactivity and the infarcted zone devoid of immunosignals became clearly visible. Concomitant GFAP-staining (B’) visualized strongly activated astrocytes with numerous thickened processes reaching the region with densely packed, and STL-labeled microglia/macrophages (B”), which was even better visible after merging the staining patterns (B”‘). Scale bars: in A” and B” = 100 μm, in A”‘ and B”‘ = 50 μm.

To explore the potential overlapping of aquaporin-4 and GFAP, as both formally represent astroglial markers, subsequent analyses were focused on the striatal ischemic border zone and combined with STL-staining to consider the vascular constituent of the neurovascular unit (Fig 10). At the first glance, aquaporin-4 was found to overlap in large parts of GFAP-immunopositive area (Fig 10A and 10A’), but not in the primarily ischemia-affected area as indicated by the STL-stained endothelia and immune cells (Fig 10A” and 10A”‘). At a higher magnification, the staining pattern of aquaporin-4 and GFAP was characterized by a few directly overlapping elements, but mainly aquaporin-4-positive structures, presumably representing astrocyte endfeet, that were in close regional association to astroglial somata as visualized by GFAP (Fig 10B”‘). A strong lectin-histochemical labeling was displayed by densely packed microglia/macrophages and some endothelial elements in the infarcted tissue. The overlay of all three staining patterns revealed the separated localization of immune cells with respect to both astroglial markers (Fig 10A”‘ and 10B”‘).

**Fig 11:**
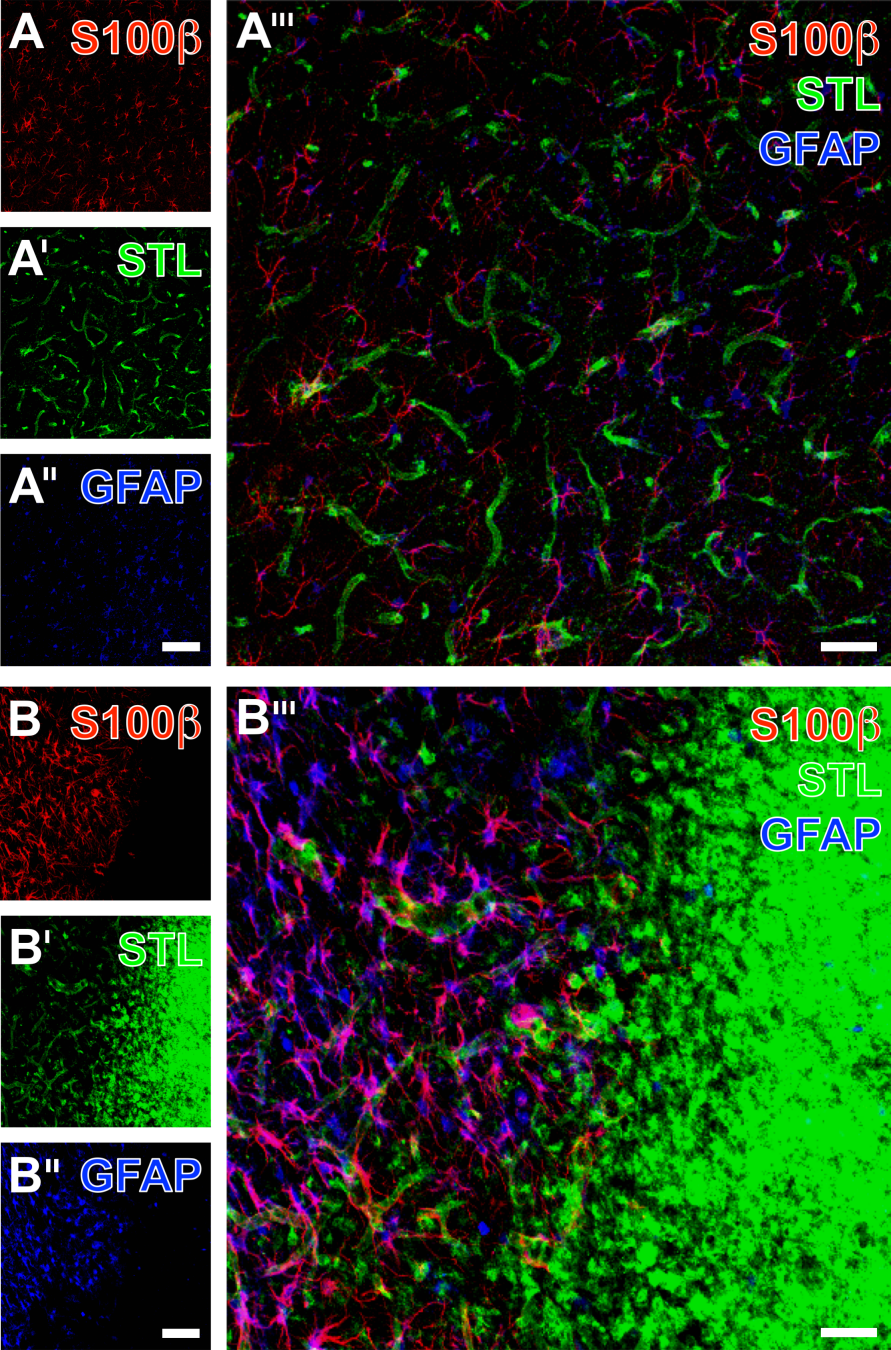
Immunofluorescence labeling of the astroglial markers S100β and GFAP combined with the lectin-histochemical staining of STL-binding sites in the non-affected *versus* the ischemia-affected neocortex from non-treated rats 7 days after transient focal cerebral ischemia. **S100β-immunoreactivity (A, B) was visible in numerous astrocytes displaying their somata in both the ischemia-affected and the naive tissue.** In contrast, anti-GFAP predominantly visualized fine astroglial processes in the non-affected region (A”), while the ischemic hemisphere was characterized by the visualization of GFAP-positive astroglial somata and processes with an activated appearance (B”). Remarkably, the ischemic region displayed numerous STL-marked microglia/macrophages (B’). The overlay of all 3 staining patterns revealed the complementary occurrence of immune cells and astroglial under ischemic conditions (B”‘). While most astrocytes co-expressed S100β and GFAP and therefore appeared purple, a few red cells were obviously mono-labeled with anti-S100β. Scale bar in A” (also valid for A and A’) = 100 μm, in B” (also valid for B and B’) = 100 μm, and in A”‘ and B”‘ = 50 μm.

In order to investigate the different patterns of astroglial immunoreaction in more detail, immunolabeling of S100β as a further marker for astrocytes were combined with GFAP- and STL-staining in the ischemia-affected neocortex compared to the contralateral, non-affected hemisphere (Fig 11). Thereby, anti-S100β was found to visualize predominantly astroglial somata in both the ischemia-affected and the control region (Fig 11A and 11B). In contrast, GFAP-immunolabeling even more corresponds to astroglial processes in the non-affected region (Fig 11A”), while in the ischemic area (Fig 11B”) somata and processes displayed an overall activated appearance. With respect to the location of glial reaction, co-staining with STL (Fig 11A’ and 11B’) demonstrated that both glial markers visualized immunopositive cellular structures in close regional relationship to–but not within–the area that is drastically affected by ischemia as detected by a strong accumulation of STL-stained microglia/macrophages (right part in Fig 11B”‘).

### Vascular and immune-mediated reactions due to ischemia and EGF treatment

**Fig 12:**
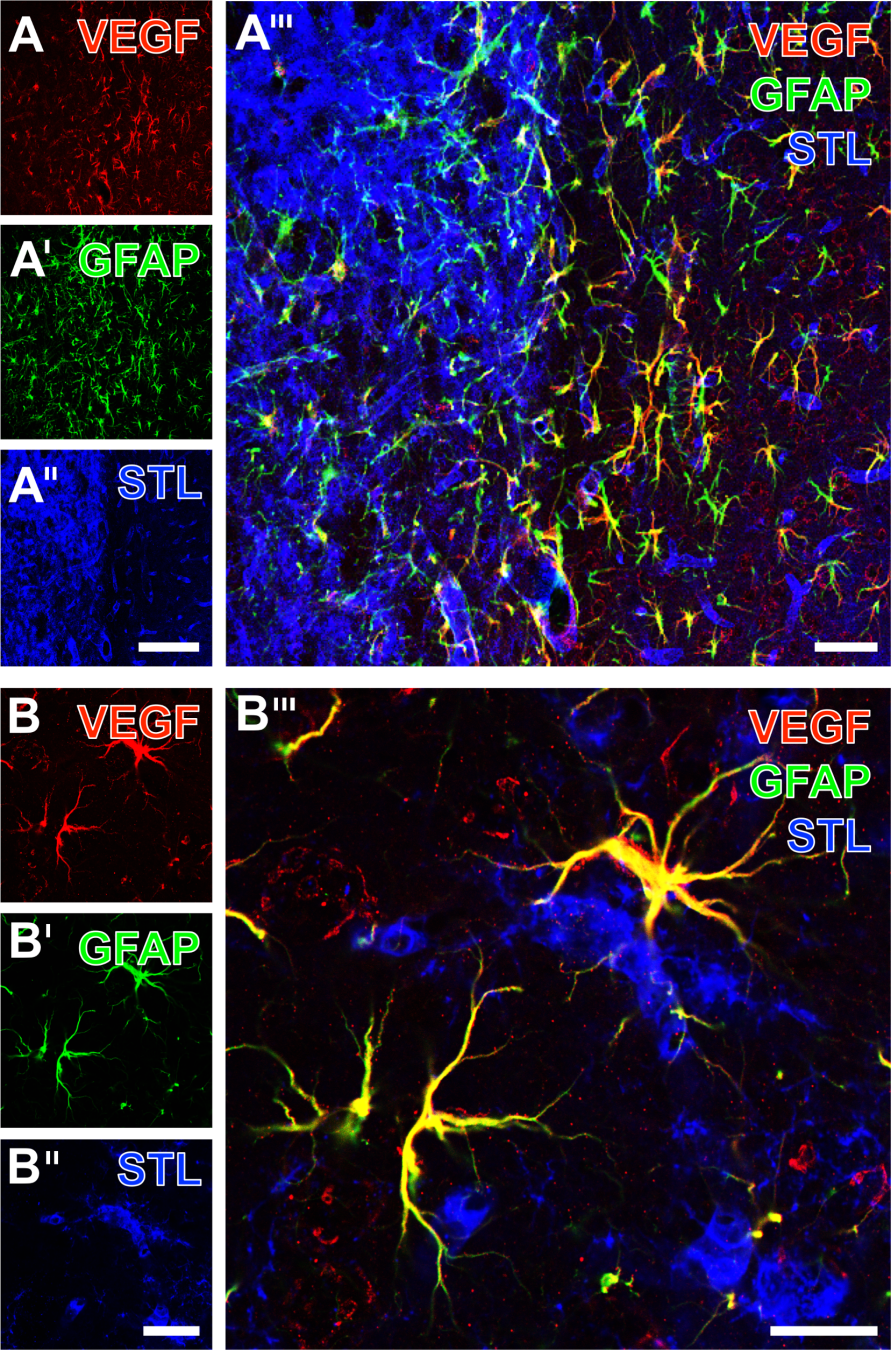
**Immunofluorescence labeling of the vascular endothelial growth factor (VEGF) combined with GFAP- and STL-staining 7 days after transient focal cerebral ischemia in the EGF-treated (A-A”‘) and in the control group (B-B”‘).** At the striatal border zone, the VEGF-staining appeared most prominent in cells with an activated morphological appearance, which also displayed astroglial GFAP-immunolabeling (A”‘). Concomitantly, STL-binding sites (A”) were seen in allocated vessels, whereas the endothelial cells in infarcted striatal tissue were dominated by allocated microglia/macrophages. The merged staining patterns (A”‘) elucidated those regions filled with immune cells surrounded by numerous, yellowish appearing activated astrocytes co-expressing VEGF and GFAP. At a higher magnification of the striatum from a control animal, immunolabeling of VEGF (B) and GFAP (B’) revealed somata and fine processes of astrocytes in the vicinity of the infarcted region. Concomitant STL-staining (B”) predominantly detected endothelial cells, and the merger of staining patterns demonstrated the close regional association of astroglial processes and vessels. Scale bars: in A” (also valid for A and A’) = 150 μm, in A”‘ = 50 μm, in B” (also valid for B and B’) = 25 μm, and in B”‘ = 15 μm.

Subsequent staining experiments were focused on the vascular endothelial growth factor (VEGF) that became interesting because of its involvement in post-ischemic processes, potentially influenced by the applied experimental treatment with neurotrophic factors. Triple fluorescence labeling of VEGF, GFAP and STL in the ischemia-affected striatum 7 days after EGF treatment revealed the allocation of VEGF and GFAP (Fig 12A and 12A”) that was also observed in a control animal (Fig 12B and 12B’). While focusing on the ischemic border zone (middle part of Fig 12A and 12A’), which became clearly demarcated by the ischemia-associated activation and up-regulation of STL-stained microglia/macrophages and endothelial structures, respectively (Fig 12A”), the VEGF-staining appeared most prominent in GFAP-immunopositive astroglia. At a higher magnification, immunolabeling of VEGF and GFAP indicated a nearly complete matching pattern, indicating that in addition to the somata also fine astroglial processes are immunopositive for VEGF (Fig 12B”‘). The overlay further elucidated that the performed STL-staining demonstrating endothelial cells to be in close vicinity to the patterns of co-expressing VEGF and GFAP (including fine astrocytic processes and endfeet), but without any evidence of full overlapping (Fig 12B”‘).

**Fig 13:**
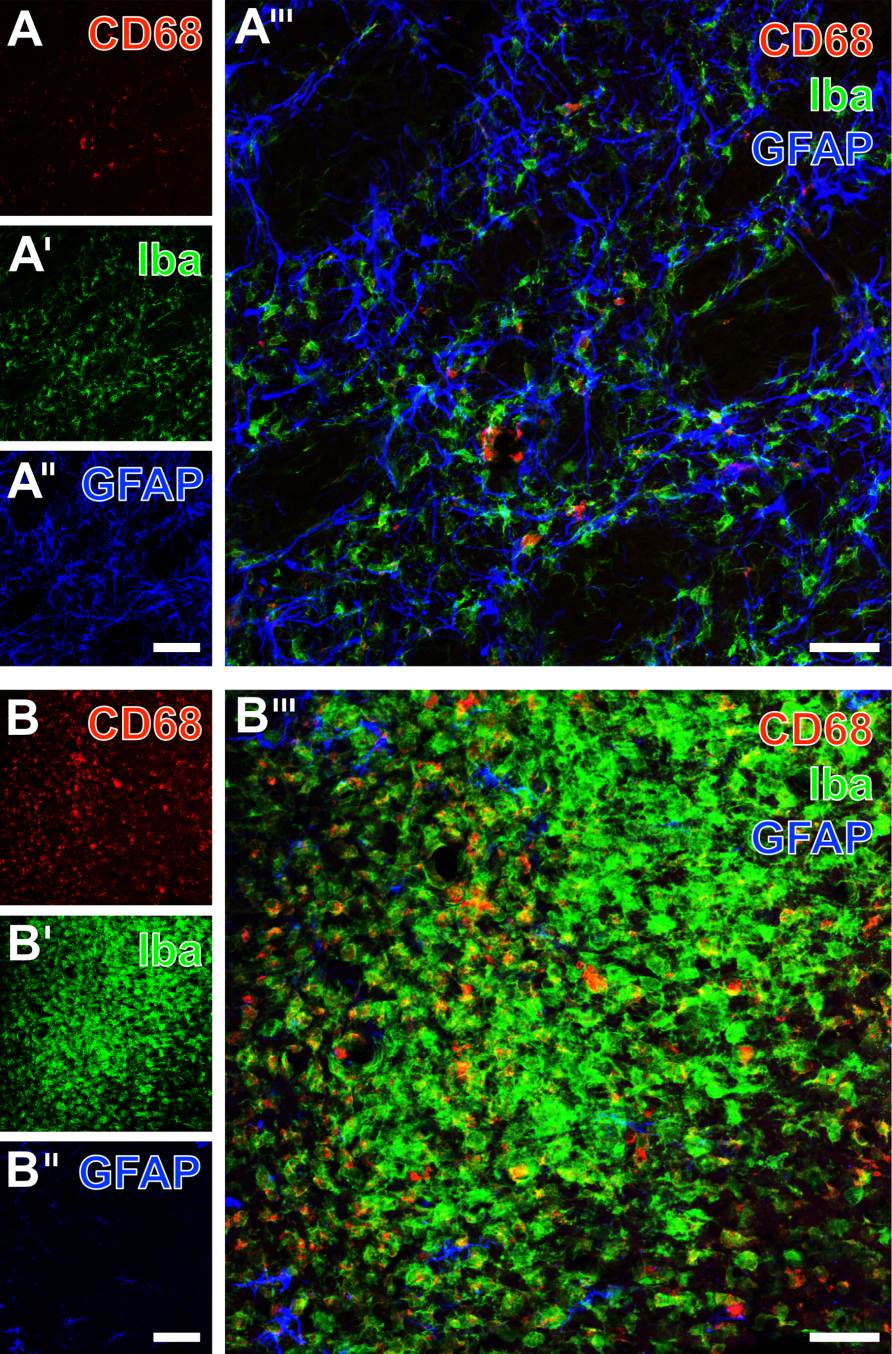
**Fluorescence labeling of the immune cell markers CD68 and Iba combined with GFAP-immunodetection 7 days after focal cerebral ischemia in the striatum from an animal of the EGF group (A-A”‘) and the neocortex from a rat of the control group (B-B”‘).** The macrophage marker CD68 (A) was observed in several, mostly ameboid cells in the infarcted tissue, while Iba-immunoreactivity (A’) was seen in many more microglia/macrophages. Both markers appeared embedded in a network of activated GFAP-stained astroglia (A”), which became even clearer by merging the staining patterns (A”‘). In the ischemia-affected neocortex, CD68-immunolabeling (B) was predominantly found in numerous ameboid cells, whereas Iba (B’) was expressed in even more microglia/macrophages. Concomitant GFAP-staining (B”) revealed some activated astrocytes, but also larger infarcted areas devoid of this marker. The overlay of staining patterns (B”‘) also visualized macrophages co-expressing CD68 (red) and Iba (green), indicated by a yellow appearing rims. Scale bars: in A” and B” (also valid for A, A’, B and B’) = 100 μm, in A”‘ and B”‘ = 50.

To more closely capture immune-mediated consequences of EGF treatment, triple immunofluorescence labeling was performed including CD68 as marker for a subset of macrophages, Iba for the visualization of microglia/macrophages and astroglial GFAP. Thereby, the ischemia-affected striatum 7 days after EGF treatment comprised several CD68-positive cells (Fig 13A), embedded in the strong local reaction of microglia and astrocytes (Fig 13A’ and 13A”). While merging the staining patterns, a few, mostly ameboid cells became evident characterized by co-labeling of CD68 and Iba (Fig 13A”‘). A comparable pattern was seen in the neocortex 7 days after experimental without treatment with neurotrophic factors: Several CD68-positive cells (Fig 13B) were embedded in numerous Iba-positive microglia/macrophages (Fig 13B’), while the merging of patterns indicated a few cells co-expressing CD68 and Iba (Fig 13B”‘).

## Discussion

The present study focused on cellular alterations 7 days after experimental focal cerebral ischemia as visualized by multiple fluorescence labeling and completes our previous serial MRI analyses of the same animals [34]. To provide translationally relevant insights into those delayed cellular response related to a clinically significant period of focal cerebral ischemia, the widely accepted NVU concept for stroke-related tissue damage was considered and experiments subsequently addressed astroglial and microglial changes as well as the vasculature [13, 15, 21, 36]. Currently, treatment for acute ischemic stroke is focused on recanalizing approaches with either intravenous thrombolysis [3] or mechanical thrombectomy [6]. However, an increasing need for the development of potential neuroprotective (co-)treatments with beneficial effects on post-stroke restoration has been emerged [21, 23, 37]. Consequently, the present study employed a transient model of focal cerebral ischemia, resembling successful recanalization with rapid reperfusion, and analyses were extended to experimental treatment with neurotrophic factors, believed to be critically involved in maintaining neuronal survival and regenerative processes [27, 29]. To consider translational aspects, histochemical analyses with subsequent quantifications were generally performed in an investigator-blinded fashion to the experimental group, driven by the intention to avoid any bias that might originate from expectations related to the performed experimental treatment with neurotrophic factors.

### Ischemia-related changes within the NVU in untreated animals

The importance of the cerebral endothelium as the functionally effective part of the BBB was extensively highlighted under ischemic conditions [38, 39]. From the perspective of the NVU concept, the cerebral vasculature and more specifically the endothelium responds to any alteration in the neuro-endocrine environment. Therefore, in a first set of experiments, histochemical analyses were focused on the BBB integrity in ischemia-affected brain areas and revealed a strong leakage signal of endogenous albumin indicating significant BBB breakdown as a consequence of the applied model of transient focal cerebral ischemia. With reference to our previous work using MRI to address the time course of BBB opening after experimental stroke [12] and comparable results from the fluorometric analyses done by Belayev and colleagues [11], these data support the formerly described existence of a second, relevant long-lasting effect on the BBB integrity beyond the 24-hour time window. Consequently, the time point of 7 days after the ischemic event used in the present study appears suitable to investigate the potential long-term influence of early experimental treatments targeting the NVU.

While focusing on morphological alterations due to the ischemic stimulus, we observed–at least in part–thinned endothelia, which is in line with findings from Taguchi et al. [40] and Morris et al. [41]. These authors had demonstrated a decreased diameter of ischemia-affected capillaries and microvessels in diverse brain regions following global brain ischemia. With reference to the associated basal membrane, we found a strong up-regulation of collagen IV due to transient MCA occlusion, indicating significant cellular alterations also in the delayed phase (i.e. 7 days) after experimental stroke. Given the fact that basement membranes play a pivotal role in stabilizing the vasculature and are involved in signaling processes with the associated extracellular matrix [39, 42], the observed up-regulation of collagen IV suggests highly dynamic actions at this stage after ischemia within the NVU. Recently, the functional relevance of glial cells in close regional association to the vasculature has been highlighted in several reports, indicating that a normal function of astrocytes, especially their endfeet as well as the microglia are essential for maintaining homeostasis within the NVU [39, 43, 44].

Ischemia-related morphological changes of astrocytes have been studied in diverse scenarios: Kwon and colleagues investigated the basement membrane-to-astrocyte contact at several time points after permanent MCA occlusion in rats and found a significant decrease of regular contacts over time, correlating with the activity of matrix metalloproteinase-9, believed to be involved in BBB degrading processes [45]. In a further study, Ito et al. performed transient forebrain ischemia and observed swollen astrocyte endfeet during the first hours after the ischemic stimulus, while microvessels became increasingly obstructed, which led to the hypotheses of an astrocyte endfeet-mediated vascular compression during the early phase of cerebral ischemia [46]. In our study, multiple immunofluorescence labeling concomitantly demonstrated the up-regulation of collagen IV and a nearly complete loss of aquaporin-4 immunoreactivity (see Fig 8), indicating significant reaction at the area of vascular and astroglial coupling in terms of dispersed astrocyte endfeet. However, we also observed a preserved, rim-like pattern of aquaporin-IV staining in the ischemia-affected striatum suggesting highly variable situations with an intact glia limitans at the focused delay time point following transient focal cerebral ischemia. Further, GPAP as the typical marker for astroglia was found to overlap in large parts with aquaporin-IV, but not in the area of maximum tissue damage. To explore the observed inhomogeneous astroglial patterns, concomitant labeling of three astroglial markers (GFAP, aquaporin-4 and S100β) allowed the perception that aquaporin-IV was limited to astrocyte endfeet, while GFAP predominantly visualized associated processes.

In the present study we regularly observed a glial formation in terms of a scar located at the ischemic bordering zone. Notably, reactive astrogliosis has been widely described in various neurological diseases [47, 48]. Very recently, Anderson and colleagues provided evidence that glial scar formation is associated with neuronal regeneration rather than the earlier assumed inhibition of regenerative processes [49]. This perspective is in line with a previous report by Salhia et al. [50], who correlated reactive astrocytosis with the expression of the vascular endothelial growth factor (VEGF) and subsequent histochemical signs of angiogenesis. Therefore, we assessed the glial scar formation in our model of focal cerebral ischemia as a typical part of the post-stroke regenerative process, clearly demonstrated at 7 days after the ischemic event. On the other hand, VEGF derived from astrocytes has also been described to mediate BBB disruption in a mouse model of multiple sclerosis [51]. However, assuming that chronic inflammatory diseases of the central nervous system and acute pathological alterations of the vasculature in the brain are characterized by–at least in part–comparable mechanisms of BBB leakage, different consequences of reduced BBB integrity with respect to the biphasic nature after cerebral ischemia need to be discussed. In light of the transition process from injury to repair, described as the new penumbra by Lo [23], the first wave of BBB opening could be assumed to have deleterious effects, whereas the second wave might me associated with regenerative properties [34]. Overall, these insights qualify astrocytes and their secreted factors–especially VEGF–as promising neuroprotective targets within the NVU [52].

Concerning morphological changes of microglia, histochemical analyses revealed a strong effect in terms of a distinct activated appearance in the area of strongest ischemic tissue damage. Considering the variety of immunoactive cell types in the brain, multiple timely orchestrated and dynamic interactions against endogenous and exogenous stimuli including ischemia can be perceived [53, 54]. The observed histochemical alterations in our study indicated a strong ischemia-related activation of a relatively inhomogeneous cell population as visualized by increased Iba-immunoreactivity. While the functional contribution of microglia within the NVU is part of an ongoing discussion, it is well known that they exhibit the potential to promote both deleterious and regenerative actions, depending for instance on the time since the ischemic stimulus as well as on the individual age and on their location in the brain [54, 55].

Considering regional aspects of experimental focal cerebral ischemia, we performed various analyses focusing on (1) the ischemic core within the striatal region that is primarily affected by the filament-based ischemia model, (2) the ischemic border zone as well as (3) the contralateral and thereby non-affected region as control (see Fig 5). Investigating ischemia-related tissue damage based on the NVU concept, these regions were quantitatively analyzed for alterations of collagen IV, GFAP and Iba. Thereby, the staining intensity for collagen IV and Iba was the strongest in the ischemic core with a remarkably decreasing course towards the border zone and the control region. As a confirmation of our qualitative findings, GFAP was observed most prominently in the ischemic bordering zone, while the ischemic core was nearly devoid of any immunosignal. These data are in excellent accordance with the aforementioned astrocyte scar formation [49, 56].

Concerning translational aspects, it is important to note that reactive astrogliosis in close regional association to the ischemic lesion–mostly described at the peri-infarct region–was robustly demonstrated after experimental stroke [57, 58], and also in brain tissues of stroke patients [59]. Together with the addressed vascular and microglial constituents of the NVU, the strong, simultaneous and region-specific up-regulation of collagen IV, Iba and GFAP at day 7 after the ischemic event clearly indicated an ongoing cellular effect at this time stage. While considering the overall existing highly significant p-values and consistent data for both histochemical endpoints, i.e. staining density and intensity, these findings appeared very robust and allowed subsequent analyses of experimental treatments in the midst of the transition process from acute damage to regenerative processes.

### Cellular responses to experimental treatment with neurotrophic factors

As neurotrophic may represent potent and safe co-treatments for ischemic stroke, our study also addressed their timely effects on diverse cellular components within the NVU while applying the same regions and histochemical endpoints as for the quantitative analyses in the overall sample. The intention to perform these histological investigations mainly resulted from our earlier report [34], which described a long-lasting reduction of the ischemic lesion volume after single and early treatment of experimental stroke with PEDF and EGF. Remarkably, in this study PEDF was also found to stabilize the BBB integrity at 24 hours and at day 7 after the ischemic event [34], which was ascribed to anti-oxidative effects and a protective role for diverse tight junction proteins [60, 61].

In the present study, treatment with PEDF resulted in attenuated up-regulation of collagen IV in subcortical regions affected by ischemia, while GFAP and Iba were not affected. Interestingly, an earlier study by Zhang and colleagues provided evidence that PEDF significantly influenced the activity of the vascular endothelial growth factor (VEGF) in terms of a down-regulation of the VEGF expression *via* the transcriptional level [61]. Conclusively, a further study indicated that early treatment with an anti-VEGF antibody resulted in BBB stabilization in a rat model of focal cerebral ischemia [62], which led to the hypothesis that VEGF is critically involved in maintaining BBB integrity. Indeed, Zhang et al. convincingly demonstrated in a rat model of permanent focal cerebral ischemia that treatment with recombinant human VEGF in the early phase (1 hour after ischemic onset) can lead to BBB opening, while delayed administration (48 hours after ischemia onset) can result in an increased angiogenesis at the peri-infarct region [63]. Consequently, our findings together with these earlier studies lead to the hypothesis that the two waves of BBB opening following ischemic stroke could be positively influenced by early PEDF treatment. Concerning the potential underlying mechanisms, VEGF likely inhibits the BBB breakdown as a typical feature during the early phase after the ischemic event and promotes angiogenesis in later stages, counteracting tissue damage and leaky vessels. In line with this perspective, the PEDF-related attenuation of usually up-regulated collagen IV after ischemia might be considered as a stabilizing effect on the vasculature.

To further explore our finding that treatment with EGF was devoid of a significant effect on collagen IV, multiple immunofluorescence labeling was performed which allowed simultaneous visualization of VEGF, astrocytes and the endothelium under ischemic conditions. The staining patterns of mainly astroglia-associated VEGF did not differ visually between the EGF-treated and the control group (see Fig 12). Albeit this qualitative finding is devoid of a quantitative confirmation, it might be concluded hat the earlier reported neuroprotective effects of EGF in a histological work [33] as well as a MRI-based study [34] probably not result *via* VEGF and vessel-associated changes. This perspective is further supported by the performed correlation analyses between changes on collagen IV and GFAP (see Fig 7), providing no evidence for a statistical relationship–neither in the overall sample nor in the respective treatment groups. However, as neuroprotective effects due to anti-oxidative properties were well described for other neurotrophic factors, e.g., the nerve growth factor [64], EGF probably also counteracts the ischemia-related oxidative stress. Notably, our quantitative analyses of glial alterations depending on experimental treatment with EGF and PEDF (see Fig 5) revealed a significantly different GFAP-immunoreactivity between both neurotrophic factors, while EGF treatment resulted in a decreased immunoreactivity when compared to PEDF. This finding might indicate that the neuroprotective effect of EGF on infarct lesion size [34] is more related to astroglial than to vascular changes. However, since no significantly differing GFAP-immunoreactivity was found after comparing the control and the EGF group, the functional relevance of observed changes remained unclarified at this stage, and needs to be addressed by further studies.

In the present study, treatment with neurotrophic factors did not result in a significant effect on the immunologically active population of microglia/macrophages (see bottom of Fig 5). Subsequent multiple immunofluorescence labeling with Iba and CD68 –as an additional marker of macrophages–confirmed the quantitative data by a comparable pattern of overlapping cellular structures when separated for the control and the EGF-treated group.

### Methodological considerations

The present study has some limitations. First, histochemical analyses were limited to a single time point (i.e. day 7 after transient focal cerebral ischemia), which has been chosen to investigate delayed cellular alterations after the ischemic event, and a single dosage of PEDF and EGF, respectively. This time point was chosen as the first week after ischemia onset might represent a time frame that includes damaging events and the initiation of regenerative processes in parallel [8], which were both addressed by the present study. Second, early treatment with EGF and especially PEDF was shown to result in smaller infarct volumes and changes over time in MRI-based analyses [34], whereas histological differences between treatment groups were found to a much lesser degree. Although investigations by MRI and histochemical methods might provide a generally restricted comparability, future studies are needed to address cellular reactions at earlier time points after experimental stroke to verify the MRI findings as shown in our previous work [34]. Third, efforts have been made to use a broad spectrum of widely accepted markers for the histochemical detection of various NVU components. Additional studies might comprise even more vascular and glial markers, added by quantitative data on fluorescence labeling and the respective marker expression to allow the distinction of effects related to an enhanced immunoreactivity *versus* increased levels of cellular components. However, the present study was intended as a translational oriented investigation considering a reperfusion scenario of an initially occluded middle cerebral occlusion to mimic current efforts on clinical stroke therapy, and a sample size that allowed quantitative analyses of diverse cellular constituents related to the widely accepted NVU concept.

## Conclusions

Despite the given limitations, the present study revealed new insights in delayed cellular alterations after 60 minutes of focal cerebral ischemia considering the penumbra and NVU concept as today’s most sophisticated models for stroke-related tissue damage. Multiple fluorescence labeling revealed significant BBB breakdown associated with a strong up-regulation of collagen IV as a basement membrane constituent and histochemical signs of microglia activation and degraded astrocyte endfeet in ischemia-affected areas. In contrast, activated astroglia was found to form a scar-like pattern in the ischemic border zone. Treatment with PEDF administered during the early phase after the ischemic event resulted in a significant attenuation of the usually observed up-regulation of the collagen IV-immunoreactivity, indicating a vessel-mediated mode of action concerning the previously demonstrated neuroprotective and BBB stabilizing effects. However, experimental treatment with neurotrophic factors did not affect the delayed astroglial and microglial reaction.

In summary, the emerging details on delayed vascular pathology and glial reactions may allow more specific treatment interventions for acute ischemic stroke, thus focusing on vasculature elements as for instance the basal membrane and associated structures like astrocyte endfeet. Future studies are needed to further investigate the effects of experimental co-treatment with neurotrophic factors in more detail, primarily to verify the observed effects of PEDF on the vasculature in the setting of recanalizing approaches, which might allow enhanced regeneration after acute ischemic stroke.

## Supporting information

S1 File Supporting information_dataset. This file includes the complete dataset of original measurements underlying the study.
(CSV)

S2 File Supporting information_syntax. This file includes of the original SPSS syntax that was used for calculations throughout the manuscript.
(PDF)
