# Supplementary material for: Delayed histochemical alterations within the neurovascular unit due to transient focal cerebral ischemia and experimental treatment with neurotrophic factors
Source: PLoS One. 2017 Apr 26;12(4):e0174996. doi: 10.1371/journal.pone.0174996 (PMC5405989; doi:10.1371/journal.pone.0174996)
Supplement: S2 File — This file includes of the original SPSS syntax that was used for calculations throughout the manuscript. (PDF) [file pone.0174996.s002.pdf]

```
* Encoding: UTF-8.

*****************************************************************
**** gerundete Mittelwerte f�r Dichte:

compute GFAP_infEx_Dichte =rnd(mean.3(GFAP_S1_InfEx_Dichte,
GFAP_S2_InfEx_Dichte, GFAP_S3_InfEx_Dichte)).
compute GFAP_infper_Dichte =rnd(mean.3(GFAP_S1_Infper_Dichte,
GFAP_S2_Infper_Dichte, GFAP_S3_Infper_Dichte)).
compute GFAP_infzent_Dichte =rnd(mean.3(GFAP_S1_Infzent_Dichte,
GFAP_S2_Infzent_Dichte, GFAP_S3_Infzent_Dichte)).
compute GFAP_cort_Dichte =rnd(mean.3(GFAP_S1_cort_Dichte,
GFAP_S2_cort_Dichte, GFAP_S3_cort_Dichte)).
des GFAP_infEx_Dichte GFAP_infper_Dichte GFAP_infzent_Dichte
GFAP_cort_Dichte.

compute Iba_infEx_Dichte =rnd(mean.3(Iba_S1_InfEx_Dichte,
Iba_S2_InfEx_Dichte, Iba_S3_InfEx_Dichte)).
compute Iba_infper_Dichte =rnd(mean.3(Iba_S1_Infper_Dichte,
Iba_S2_Infper_Dichte, Iba_S3_Infper_Dichte)).
compute Iba_infzent_Dichte =rnd(mean.3(Iba_S1_Infzent_Dichte,
Iba_S2_Infzent_Dichte, Iba_S3_Infzent_Dichte)).
compute Iba_cort_Dichte =rnd(mean.3(Iba_S1_cort_Dichte,
Iba_S2_cort_Dichte, Iba_S3_cort_Dichte)).
des Iba_infEx_Dichte Iba_infper_Dichte Iba_infzent_Dichte
Iba_cort_Dichte.

compute Koll_infEx_Dichte =rnd(mean.3(Koll_S1_InfEx_Dichte,
Koll_S2_InfEx_Dichte, Koll_S3_InfEx_Dichte)).
compute Koll_infper_Dichte =rnd(mean.3(Koll_S1_Infper_Dichte,
Koll_S2_Infper_Dichte, Koll_S3_Infper_Dichte)).
compute Koll_infzent_Dichte =rnd(mean.3(Koll_S1_Infzent_Dichte,
Koll_S2_Infzent_Dichte, Koll_S3_Infzent_Dichte)).
compute Koll_cort_Dichte =rnd(mean.3(Koll_S1_cort_Dichte,
Koll_S2_cort_Dichte, Koll_S3_cort_Dichte)).
des Koll_infEx_Dichte Koll_infper_Dichte Koll_infzent_Dichte
Koll_cort_Dichte.

**** Differenzen f�r Dichte:

compute GFAP_Diff_infzent_Infex_Dichte = GFAP_infzent_Dichte -
GFAP_infex_Dichte.
compute GFAP_Diff_Infper_Infex_Dichte = GFAP_infper_Dichte -
GFAP_infex_Dichte.
compute GFAP_Diff_infzent_infper_Dichte = GFAP_infzent_Dichte -
GFAP_infper_Dichte.
des GFAP_Diff_infzent_Infex_Dichte GFAP_Diff_Infper_Infex_Dichte
GFAP_Diff_infzent_infper_Dichte.

compute Iba_Diff_infzent_Infex_Dichte = Iba_infzent_Dichte -
Iba_infex_Dichte.
compute Iba_Diff_Infper_Infex_Dichte = Iba_infper_Dichte -
Iba_infex_Dichte.
compute Iba_Diff_infzent_infper_Dichte = Iba_infzent_Dichte -
```

```
Iba_infper_Dichte.
des Iba_Diff_infzent_Infex_Dichte Iba_Diff_Infper_Infex_Dichte
Iba_Diff_infzent_infper_Dichte.

compute Koll_Diff_infzent_Infex_Dichte = Koll_infzent_Dichte -
Koll_infex_Dichte.
compute Koll_Diff_Infper_Infex_Dichte = Koll_infper_Dichte -
Koll_infex_Dichte.
compute Koll_Diff_infzent_infper_Dichte = Koll_infzent_Dichte -
Koll_infper_Dichte.
des Koll_Diff_infzent_Infex_Dichte Koll_Diff_Infper_Infex_Dichte
Koll_Diff_infzent_infper_Dichte.

**** gerundete Mittelwerte f�r Intensit�t:

compute GFAP_infEx_Intens =mean.3(GFAP_S1_InfEx_Intens,
GFAP_S2_InfEx_Intens, GFAP_S3_InfEx_Intens).
compute GFAP_infper_Intens =mean.3(GFAP_S1_Infper_Intens,
GFAP_S2_Infper_Intens, GFAP_S3_Infper_Intens).
compute GFAP_infzent_Intens =mean.3(GFAP_S1_Infzent_Intens,
GFAP_S2_Infzent_Intens, GFAP_S3_Infzent_Intens).
compute GFAP_cort_Intens =mean.3(GFAP_S1_cort_Intens,
GFAP_S2_cort_Intens, GFAP_S3_cort_Intens).
des GFAP_infEx_Intens  GFAP_infper_Intens GFAP_infzent_Intens
GFAP_cort_Intens.

compute Iba_infEx_Intens =mean.3(Iba_S1_InfEx_Intens,
Iba_S2_InfEx_Intens, Iba_S3_InfEx_Intens).
compute Iba_infper_Intens =mean.3(Iba_S1_Infper_Intens,
Iba_S2_Infper_Intens, Iba_S3_Infper_Intens).
compute Iba_infzent_Intens =mean.3(Iba_S1_Infzent_Intens,
Iba_S2_Infzent_Intens, Iba_S3_Infzent_Intens).
compute Iba_cort_Intens =mean.3(Iba_S1_cort_Intens,
Iba_S2_cort_Intens, Iba_S3_cort_Intens).
des Iba_infEx_Intens Iba_infper_Intens  Iba_infzent_Intens
Iba_cort_Intens.

compute Koll_infEx_Intens =mean.3(Koll_S1_InfEx_Intens,
Koll_S2_InfEx_Intens, Koll_S3_InfEx_Intens).
compute Koll_infper_Intens =mean.3(Koll_S1_Infper_Intens,
Koll_S2_Infper_Intens, Koll_S3_Infper_Intens).
compute Koll_infzent_Intens =mean.3(Koll_S1_Infzent_Intens,
Koll_S2_Infzent_Intens, Koll_S3_Infzent_Intens).
compute Koll_cort_Intens =mean.3(Koll_S1_cort_Intens,
Koll_S2_cort_Intens, Koll_S3_cort_Intens).
des Koll_infEx_Intens Koll_infper_Intens Koll_infzent_Intens
Koll_cort_Intens.

**** Differenzen f�r Intensit�t:

compute GFAP_Diff_infzent_Infex_Intens = GFAP_infzent_Intens -
GFAP_infex_Intens.
compute GFAP_Diff_Infper_Infex_Intens = GFAP_infper_Intens -
GFAP_infex_Intens.
```

```
compute GFAP_Diff_infzent_infper_Intens = GFAP_infzent_Intens -
GFAP_infper_Intens.
des GFAP_Diff_infzent_Infex_Intens GFAP_Diff_Infper_Infex_Intens
GFAP_Diff_infzent_infper_Intens.

compute Iba_Diff_infzent_Infex_Intens = Iba_infzent_Intens -
Iba_infex_Intens.
compute Iba_Diff_Infper_Infex_Intens = Iba_infper_Intens -
Iba_infex_Intens.
compute Iba_Diff_infzent_infper_Intens = Iba_infzent_Intens -
Iba_infper_Intens.
des Iba_Diff_infzent_Infex_Intens Iba_Diff_Infper_Infex_Intens
Iba_Diff_infzent_infper_Intens.

compute Koll_Diff_infzent_Infex_Intens = Koll_infzent_Intens -
Koll_infex_Intens.
compute Koll_Diff_Infper_Infex_Intens = Koll_infper_Intens -
Koll_infex_Intens.
compute Koll_Diff_infzent_infper_Intens = Koll_infzent_Intens -
Koll_infper_Intens.
des Koll_Diff_infzent_Infex_Intens Koll_Diff_Infper_Infex_Intens
Koll_Diff_infzent_infper_Intens.


******************************************


DATASET ACTIVATE DatenSet1.
DESCRIPTIVES VARIABLES=GFAP_infEx_Dichte GFAP_infper_Dichte
GFAP_infzent_Dichte GFAP_cort_Dichte Iba_infEx_Dichte
Iba_infper_Dichte Iba_infzent_Dichte Iba_cort_Dichte
Koll_infEx_Dichte Koll_infper_Dichte Koll_infzent_Dichte
Koll_cort_Dichte
  /STATISTICS=MEAN STDDEV MIN MAX SEMEAN.


DATASET ACTIVATE DatenSet1.
DESCRIPTIVES VARIABLES=GFAP_infEx_Intens  GFAP_infper_Intens
GFAP_infzent_Intens  GFAP_cort_Intens Iba_infEx_Intens
Iba_infper_Intens  Iba_infzent_Intens Iba_cort_Intens
Koll_infEx_Intens Koll_infper_Intens Koll_infzent_Intens
Koll_cort_Intens
  /STATISTICS=MEAN STDDEV MIN MAX SEMEAN.


NPAR TESTS
  /WILCOXON=Koll_infzent_Intens Koll_infzent_Intens
Koll_infzent_Intens WITH Koll_infper_Intens
    Koll_cort_Intens Koll_infEx_Intens (PAIRED)
  /MISSING ANALYSIS.


NPAR TESTS
  /K-W=Koll_Diff_infzent_infper_Intens BY Gruppe(0 2)
```

```
  /MISSING ANALYSIS.


DATASET ACTIVATE DatenSet1.
NPAR TESTS
  /WILCOXON=Koll_infzent_Intens Koll_infzent_Intens
Koll_infper_Intens GFAP_infzent_Intens
    GFAP_infzent_Intens GFAP_infper_Intens Iba_infzent_Intens
Iba_infzent_Intens Iba_infper_Intens WITH
    Koll_infper_Intens Koll_infEx_Intens Koll_infEx_Intens
GFAP_infper_Intens GFAP_infEx_Intens
    GFAP_infEx_Intens Iba_infper_Intens Iba_infEx_Intens
Iba_infEx_Intens (PAIRED)
  /STATISTICS DESCRIPTIVES
  /MISSING ANALYSIS.


NPAR TESTS
  /WILCOXON=Koll_infzent_Dichte Koll_infzent_Dichte
Koll_infper_Dichte GFAP_infzent_Dichte
    GFAP_infzent_Dichte GFAP_infper_Dichte Iba_infzent_Dichte
Iba_infzent_Dichte Iba_infper_Dichte WITH
    Koll_infper_Dichte Koll_infEx_Dichte Koll_infEx_Dichte
GFAP_infper_Dichte GFAP_infEx_Dichte
    GFAP_infEx_Dichte Iba_infper_Dichte Iba_infEx_Dichte
Iba_infEx_Dichte (PAIRED)
  /STATISTICS DESCRIPTIVES
  /MISSING ANALYSIS.

DATASET ACTIVATE DatenSet1.
DESCRIPTIVES VARIABLES=Koll_Diff_infzent_Infex_Intens
Koll_Diff_infzent_Infex_Dichte GFAP_Diff_Infper_Infex_Intens
GFAP_Diff_Infper_Infex_Dichte Iba_Diff_infzent_Infex_Dichte
Iba_Diff_infzent_Infex_Intens
  /STATISTICS=MEAN STDDEV MIN MAX SEMEAN.

temp.
select if gruppe = 0.
DESCRIPTIVES VARIABLES=Koll_Diff_infzent_Infex_Intens
Koll_Diff_infzent_Infex_Dichte GFAP_Diff_Infper_Infex_Intens
GFAP_Diff_Infper_Infex_Dichte Iba_Diff_infzent_Infex_Dichte
Iba_Diff_infzent_Infex_Intens
  /STATISTICS=MEAN STDDEV MIN MAX SEMEAN.

temp.
select if gruppe = 1.
DESCRIPTIVES VARIABLES=Koll_Diff_infzent_Infex_Intens
Koll_Diff_infzent_Infex_Dichte GFAP_Diff_Infper_Infex_Intens
GFAP_Diff_Infper_Infex_Dichte Iba_Diff_infzent_Infex_Dichte
Iba_Diff_infzent_Infex_Intens
  /STATISTICS=MEAN STDDEV MIN MAX SEMEAN.

temp.
select if gruppe = 2.
```

```
DESCRIPTIVES VARIABLES=Koll_Diff_infzent_Infex_Intens
Koll_Diff_infzent_Infex_Dichte GFAP_Diff_Infper_Infex_Intens
GFAP_Diff_Infper_Infex_Dichte Iba_Diff_infzent_Infex_Dichte
Iba_Diff_infzent_Infex_Intens
  /STATISTICS=MEAN STDDEV MIN MAX SEMEAN.

NPAR TESTS
  /K-W=Koll_Diff_infzent_Infex_Dichte Koll_Diff_infzent_Infex_Intens
GFAP_Diff_Infper_Infex_Dichte
    GFAP_Diff_Infper_Infex_Intens Iba_Diff_infzent_Infex_Dichte
Iba_Diff_infzent_Infex_Intens BY
    Gruppe(0 2)
  /STATISTICS DESCRIPTIVES
  /MISSING ANALYSIS
  /METHOD=EXACT TIMER(5).

NPAR TESTS
  /K-W=Koll_Diff_infzent_Infex_Dichte GFAP_Diff_Infper_Infex_Intens
BY Gruppe(0 1)
  /STATISTICS DESCRIPTIVES
  /MISSING ANALYSIS
  /METHOD=EXACT TIMER(5).

NPAR TESTS
  /K-W=Koll_Diff_infzent_Infex_Dichte GFAP_Diff_Infper_Infex_Intens
BY Gruppe(0 2)
  /STATISTICS DESCRIPTIVES
  /MISSING ANALYSIS
  /METHOD=EXACT TIMER(5).

temp.
select if gruppe = 2.
GRAPH
  /SCATTERPLOT(BIVAR)=Koll_Diff_infzent_Infex_dichte WITH
GFAP_Diff_Infper_Infex_dichte
  /MISSING=LISTWISE.


DATASET ACTIVATE DatenSet1.
CORRELATIONS
  /VARIABLES= Koll_Diff_infzent_Infex_dichte
GFAP_Diff_Infper_Infex_dichte Koll_Diff_infzent_Infex_intens
GFAP_Diff_Infper_Infex_intens.
  /PRINT=TWOTAIL NOSIG
  /MISSING=PAIRWISE.

temp.
select if gruppe = 3.
CORRELATIONS
  /VARIABLES= Koll_Diff_infzent_Infex_dichte
iba_Diff_Infzent_Infex_dichte Koll_Diff_infzent_Infex_intens
iba_Diff_Infper_Infex_intens.

correlations Koll_Diff_infzent_Infex_dichte
```

```
Koll_Diff_infzent_Infex_intens.
correlations iba_Diff_infzent_Infex_dichte
Iba_Diff_infzent_Infex_intens.
correlations gfap_Diff_infper_Infex_dichte
gfap_Diff_infper_Infex_intens.
```
